# Synbiotics, probiotics or prebiotics in infant formula for full term infants: a systematic review

**DOI:** 10.1186/1475-2891-11-81

**Published:** 2012-10-04

**Authors:** Mary N Mugambi, Alfred Musekiwa, Martani Lombard, Taryn Young, Reneé Blaauw

**Affiliations:** 1 Division of Human Nutrition, Faculty of Medicine and Health Sciences, Stellenbosch University, Stellenbosch, Western Cape, South Africa; 2 Wits Reproductive Health & HIV Institute (WRHI), Faculty of Health Sciences, University of the Witwatersrand, Johannesburg, South Africa; 3 Centre for Evidence-Based Health Care, Faculty of Medicine and Health Sciences, Stellenbosch University, Stellenbosch, Western Cape, South Africa

**Keywords:** Synbiotic, Probiotic, Prebiotic, Full term infant

## Abstract

**Background:**

Synbiotics, probiotics or prebiotics are being added to infant formula to promote growth and development in infants. Previous reviews (2007 to 2011) on term infants given probiotics or prebiotics focused on prevention of allergic disease and food hypersensitivity. This review focused on growth and clinical outcomes in term infants fed only infant formula containing synbiotics, probiotics or prebiotics.

**Methods:**

Cochrane methodology was followed using randomized controlled trials (RCTs) which compared term infant formula containing probiotics, prebiotics or synbiotics to conventional infant formula with / without placebo among healthy full term infants. The mean difference (MD) and corresponding 95% confidence intervals (CI) were reported for continuous outcomes, risk ratio (RR) and corresponding 95% CI for dichotomous outcomes. Where appropriate, meta-analysis was performed; heterogeneity was explored using subgroup and sensitivity analyses. If studies were too diverse a narrative synthesis was provided.

**Results:**

Three synbiotic studies (N = 475), 10 probiotics studies (N = 933) and 12 prebiotics studies (N = 1563) were included. **Synbiotics** failed to significantly increase growth in boys and girls. Use of synbiotics increased stool frequency, had no impact on stool consistency, colic, spitting up / regurgitation, crying, restlessness or vomiting. **Probiotics** in formula also failed to have any significant effect on growth, stool frequency or consistency. Probiotics did not lower the incidence of diarrhoea, colic, spitting up / regurgitation, crying, restlessness or vomiting. **Prebiotics** in formula did increase weight gain but had no impact on length or head circumference gain. Prebiotics increased stool frequency but had no impact on stool consistency, the incidence of colic, spitting up / regurgitation, crying, restlessness or vomiting. There was no impact of prebiotics on the volume of formula tolerated, infections and gastrointestinal microflora. The quality of evidence was compromised by imprecision, inconsistency of results, use of different study preparations and publication bias.

**Authors’ conclusions:**

There is not enough evidence to state that supplementation of term infant formula with synbiotics, probiotics or prebiotics does result in improved growth or clinical outcomes in term infants. There is no data available to establish if synbiotics are superior to probiotics or prebiotics.

## Background

The first year of life is characterized by very rapid growth. Weight increases by 115%, body length 34% and head circumference 22% [1,2]. Many full term infants lose weight after birth and take 8–10 days to regain it back. The average infant achieves a weight gain of approximately 1.1 to 1.2 kg/month during the first 6 months, slowing down to 0.4 to 0.5 kg/month during the second 6 months. Length increases by 3.5 to 3.9 cm/month during the first 4 months, slowing down to 1.8 cm/month at 6 month of age [1]. At birth average head circumference is 35 cm and increases by an estimated 12 cm during the first year of life to approximately 47 cm. A faltering head circumference has serious implications for neural growth, maturation and is diagnostic for possible problems of brain growth [2]. Monitoring growth (weight, length and head circumference) evaluates the overall health of the infant and determines adequacy of nutritional intake [1].

To promote optimum growth, development and decrease infections, probiotics, prebiotics are added to infant formula to promote an intestinal micro flora resembling that of breastfed infants [3]. The intestinal micro flora of breastfed infants have a greater concentration of bifidobacteria and fewer potentially pathogenic bacteria compared to formula fed infants. Probiotics are “live microorganisms” which when administered in adequate amounts confer a health benefit to the host [3]. The main probiotic organisms used worldwide belong to the genera Lactobacillus and Bifidobacteria and are found in the gastrointestinal micro flora [3,4]. Probiotics are consumed in the form of fermented food, dairy products, infant and toddler formula. Prebiotics are non- digestible food ingredients that benefit the host by selectively stimulating the growth and/or activity of one or a limited number of bacteria in the colon and thereby improving the host’s health [4,5]. The most widely studied prebiotics are inulin, fructooligosaccharide (FOS) and galactooligosaccharide (GOS) which are plant storage carbohydrates in vegetables, cereals and fruit. Fructooligosaccharide and inulin are added to different foods as fat and sugar replacements to improve texture or for their functional benefits [5-8].

Probiotics improve health in different ways [3,9]. The health benefits conferred by probiotic bacteria are strain specific [3,9]. Some strains increase phagocytic activity of peripheral blood leukocytes, others strains promote production of mucosal antibodies reducing the trans-mucosal transfer of antigens. This strengthens the mucosal barrier function [10-12]. Other probiotic strains increase cytokine production such as interleukin 6 (IL-6) [13]. In healthy people probiotics rarely cause disease. The risk of developing bacteraemia from ingested lactobacilli is less than 1 per 1 million users; risk of developing fungaemia (from Saccharomyces Boulardii) is less than1 per 5.6 million users [14-16]. In many studies on infants, C- reactive protein (CRP) and IL-6 have been used to diagnose the early onset of infection [17,18]. CRP is an acute phase protein, blood levels begin to rise to 10 – 1000 fold from 1 ug/ml within 4–6 hours at the onset of an infective or inflammatory process. C- reactive protein has a relatively short half-life making it useful in monitoring infection, inflammation and response to treatment [19]. IL-6 is a pro-inflammatory cytokine which stimulates the production of acute phase proteins (such as CRP) [20]. It is readily detected in serum during inflammation and indicates the presence of infection [18,19].

Adding prebiotics to formula stimulates the growth of beneficial bacteria (such as bifidobacteria, lactobacilli) in the gastrointestinal tract to levels found in breastfed infants [9,21]. As these beneficial bacteria increase, they occupy more of the “microbiological niches” in the intestine excluding pathogens. This improves the gut mucosal barrier, prevents infections with enteric pathogens or trans-located gut bacteria [22,23]. Prebiotics have a good safety record at levels found in existing food components. Flatulence or abdominal bloating is reported at doses greater than 20g / day. Abdominal cramps or diarrhoea are reported at doses greater than 50 g / day [23].

When probiotics and prebiotics are administered simultaneously, the combination is termed Synbiotics. The prebiotic in the synbiotic mixture improves the survival of the probiotic bacteria and stimulates the activity of the host’s endogenous bacteria [9,21,24,25]. The superiority of synbiotics compared to either probiotics or prebiotics have not been demonstrated. No review has examined the impact of synbiotics on clinical outcomes in formula fed term infants. Recent systematic reviews (published from 2007 to 2011) on the use of probiotics or prebiotics in term infants have focused on prevention of allergic disease and food hypersensitivity [26,27]. Reviews on children and adults focused on upper respiratory tract infections, antibiotic associated diarrhoea and acute infectious diarrhoea [28-30]. This review focused on full term infants given only infant formula with synbiotics, probiotics or prebiotics.

The Human Research Ethics Committee at the University of Stellenbosch, South Africa reviewed the protocol, ruled that all data to be collected for this review was from the public domain and was therefore exempt from ethical approval.

### Objectives

The objectives of this systematic review were:

1) To determine the effects of infant formula containing synbiotics, probiotics or prebiotics on clinical outcomes in full term infants.

2) To explore if synbiotics are superior over probiotics or prebiotics.

## Methods

### Criteria for considering studies for this review

All randomized controlled trials (RCTs), irrespective of language, which compared the use of term infant formula containing synbiotics, probiotics or prebiotics to conventional infant formula with or without placebo amongst healthy full term infants (>37 weeks gestation or ≥ 2.5 kg birth weight, age: 0–12 months, with no disease, congenital abnormality, allergy or eczema) receiving formula feeds only. Studies published as abstracts were included if sufficient information could be obtained to assess study quality and obtain relevant study findings.

### Types of outcome measures

#### *Primary outcomes*

Growth changes (assessed for entire study duration): weight gain (g/day), linear growth (cm/week, mm/month), head growth (cm/week, mm/month). *Secondary outcomes*: Tolerance to formula: Stool characteristics: frequency, consistency, diarrhoea; Gastrointestinal symptoms (incidence of colic, spitting up/ regurgitation, vomiting, crying), average formula intake (mls/day). Infections: frequency and type of infections, use of medication (antibiotic intake); Hospitalization: Number of days in hospital. Changes in GI microflora: Changes in colony forming units (cfu/g of stool) of bifidobacteria, lactobacillus post intervention, colony forming units (cfu/g of stool) of pathogens post intervention. Immune response: C- reactive protein levels (mg/dl), Interleukin 6 (IL-6) levels (mg/dl).

### Search methods for identification of studies

A literature search regardless of language was conducted on electronic databases including The Cochrane CENTRAL Register for Controlled Trials (2010), EMBASE (1980+), Scopus (1990 present), EBSCO host (1960 to 2010), PUBMED / MEDLINE (1966 to 2010), OVID (1950 to 2010), SPORTDiscus (1960 to 2010), Web of Science (1970 to 2010), Science Direct (1950 to 2010), CINAHL (1981 to 2010), Science citation index (1970 to 2010), Latin American Caribbean Health Sciences literature (LILACS) (1965 to 2010), NLMGateway (1950–1966). RCTs published in non-English language journals were translated by independent translators who were familiar with the subject matter. The search strategy used to search PUBMED is shown below. This search strategy was modified to search other electronic databases.

(synbiotic* and probiotic* OR prebiotic*) AND (FOS or fructooligosaccharide or inulin or GOS or galactooligosaccharide) AND (infant formula* OR infant feeding OR formula OR formula milk) AND (infant* or baby or babies) NOT (preterm or premature or low birth weight babies or allergy or eczema) AND (randomized controlled trial* OR controlled clinical trial* Or random allocation*) Limits: Humans.

We also conducted a hand search on abstracts of major conference proceedings such as the Pediatric Academic Society meetings from 1990 (http://www.pas-meetings.org, http://www.abstracts2view.com), cross checked references cited in RCTs and in recent reviews (published from 2005 to 2009) for additional studies not identified by electronic searches and specialty journals which were not included in any database such as Pediatrika and Chinese Journal of Microecology.

To identify on-going and unpublished trials, we contacted experts in the field, manufacturers of infant formula containing probiotics and prebiotics, we searched web sites of companies that have conducted or were conducting RCTs on probiotics and prebiotics e.g. Pfizer (http://www.pfizerpro.com/clinicaltrials), Chris Hansen Laboratory (http://www.chr-hansen.com/research_development/documentation.html). We also searched prospective trial registries such as World Health Organization (WHO) International Clinical Trials Registry Platform Search Portal (http://www.who.int/trialsearch), Clinical Trials.gov register (http://www.clinicaltrials.gov), Current Controlled Trials *metaR*egister of Controlled Trials [*mRCT*] (http://www.controlled-trials.com/mrct) and http://www.clinicaltrialresults.org.

### Selection of studies

**Figure 1:**
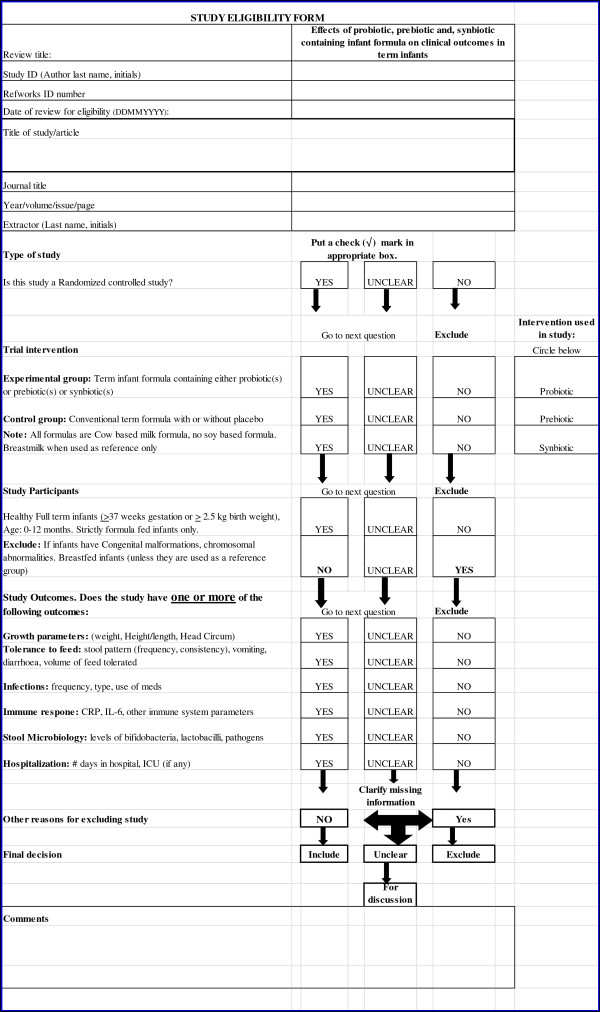
Study Eligibility form.

One reviewer (MM) independently reviewed all abstracts, citations and identified potentially eligible studies. The full reports of eligible studies were retrieved by one reviewer (MM) and the pre-specified selection criteria applied independently by two reviewers (MM, ML) using a study eligibility form (Figure 1). If more than one publication of a study existed, all reports of the study were grouped together under one study name. Any disagreements between the reviewers were resolved through discussion. Unresolved disagreements were resolved by a third party. Trial authors were contacted if eligibility was unclear.

### Assessment of quality of evidence

Two reviewers (MM, ML) independently assessed the risk of bias of included studies as described in the Cochrane Handbook for Systematic Reviews for Interventions according to the following 6 components: 1) allocation sequence generation; 2) allocation concealment; 3) blinding; 4) incomplete outcome data; 5) selective outcome reporting; and 6) other sources of bias [31]. Where necessary, trial authors were contacted for clarification on the methodology of their studies. Any disagreements regarding risk of bias were resolved through discussion between MM, ML and RB. The quality of evidence was assessed using guidelines from the Grading of Recommendations Assessment, Development and Evaluation Working Group (GRADE), http://www.gradeworkinggroup.org (accessed 2012-06-07).

### Data extraction and management

Two reviewers (MM, ML) independently extracted data using a pretested data extraction form. The reviewers (MM, ML) cross checked data and resolved any differences through discussion. One reviewer (MM) entered the data in Review Manager (RevMan 5) and the other reviewers (AM, ML) validated the data. Trial authors were contacted for missing data or for clarification.

### Data synthesis and management

Results for probiotic, prebiotic and synbiotics studies were analysed separately. No imputation measures for missing data were applied. Trial authors were contacted if there was missing data. Available case analysis was used where there was missing data. The potential impact of missing data on results is addressed in the discussion section.

Heterogeneity of the trials used in the review was assessed by visually inspecting the forest plots to detect overlapping confidence intervals and by performing a Chi^2^ test (p<0.1 was considered statistically significant because of the low statistical power of this test). An I-square test (I^2^) was also used to test for inconsistencies across studies. If the I^2^ exceeded 50% and visual inspection of the forest plot supported these results, this represented substantial heterogeneity. Since all of our meta-analyses had less than ten studies, the assessment of publication bias using funnel plots could not be done [31]. If the included studies were not clinically diverse and had similar outcome measures, a meta-analysis was carried out in Review Manager (RevMan 5) by two reviewers (AM, MM). The random effects meta-analysis model was applied to all meta-analyses since the studies were clinically heterogeneous in terms of different settings (countries), doses and strains of synbiotics, probiotics or type of prebiotics, different treatment durations, and other unforeseen factors. The inverse-variance method was used for continuous data and the Mantel-Haenszel method was used for dichotomous data. For continuous outcomes the mean difference (MD) and corresponding 95% confidence intervals (CI) were calculated. For dichotomous outcomes, the risk ratio (RR) and corresponding 95% CI were calculated. The source of statistical heterogeneity was explored using subgroup and sensitivity analyses. If studies were too diverse, no meta-analysis was conducted and a narrative synthesis was provided.

## Results

### Results of the search and description of studies

**Figure 2:**
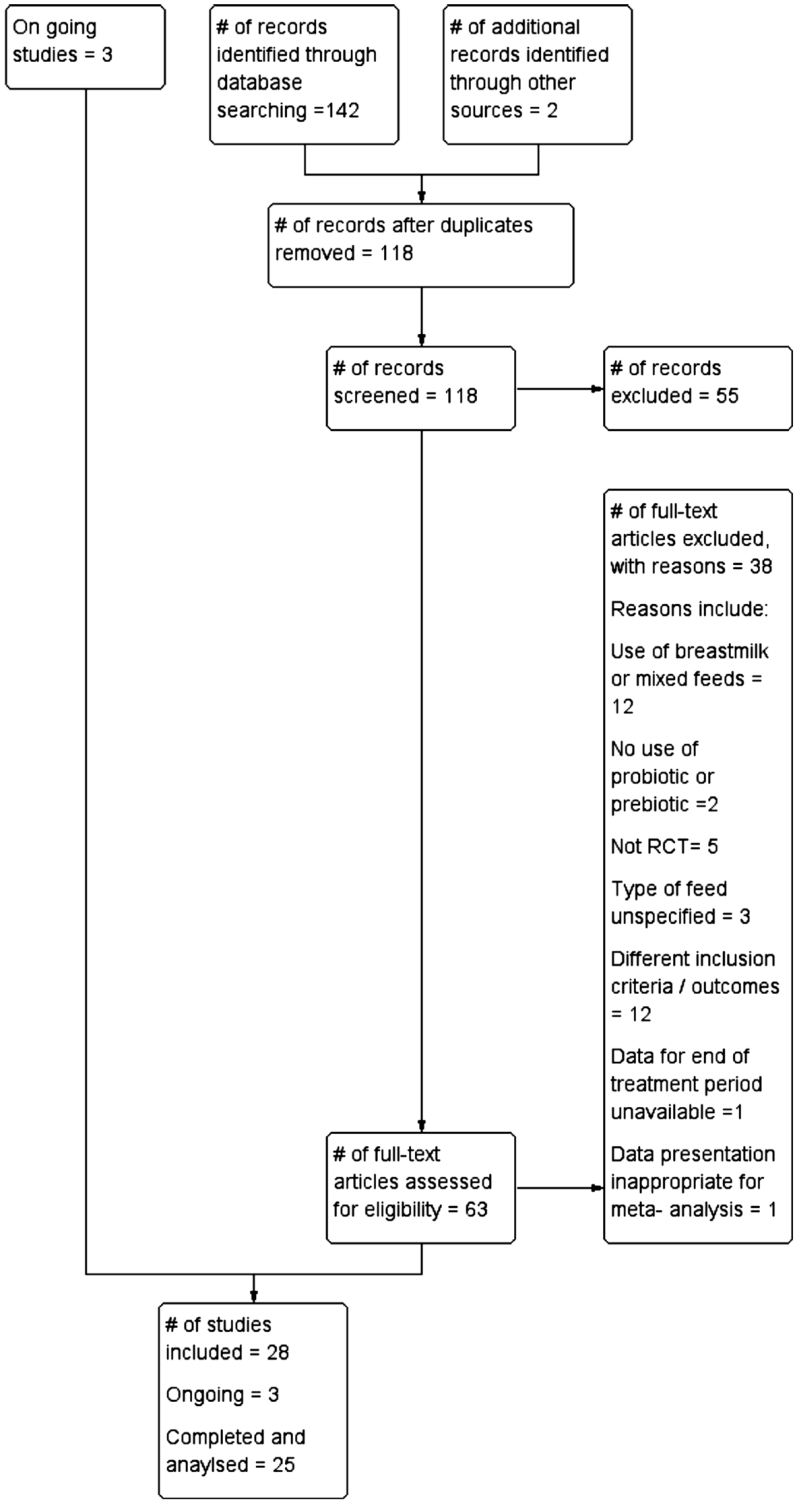
Process of study selection.

Electronic search of available databases yielded 142 citations. After reading titles and abstracts, duplicate reports were removed, 118 articles were screened and 55 articles were excluded. A hand search yielded 2 more articles. Potentially relevant full text reports were retrieved, reviewed for eligibility and a further 38 studies were excluded. One study was published in two other reports [32-34]. The three studies were considered as one study and are referred to as Moro 2006 [32]. Another study was also published in two reports; and is referred as Moro 2002 [35,36]. Twenty five studies (3 synbiotic, 10 probiotic and 12 prebiotic studies) and three on-going studies were included in this review [21,24,25,37-56]. The selection process is shown in Figure 2. Table 1 gives a list of 38 studies which were excluded for: use of breast milk or mixed feeds (12 studies), no use of probiotic or prebiotic (2 studies), being a cross over study, not RCT (5 studies), type of feed was unspecified (3 studies), different inclusion criteria or outcomes (12 studies), no data available for end of treatment period (1 study) and data presentation inappropriate for meta- analysis (3 studies) [57-94]. No eligible studies were excluded for failure to report the review’s pre-specified outcomes.

**Table 1:** Excluded studies, with reasons for exclusion

**Reasons for exclusion of studies**								
**Use of breast milk or mixed feeds (breast milk, formula, other milk – cow, buffalo, goat milk)**		**No use of probiotic, prebiotic**	**Cross over trial / study, Not RCT**	**Type of feed not clear / specified**	**Different inclusion criteria or outcomes**		**Data for end of treatment period not available**	**Data presentation inappropriate for Meta -analysis**
Allen 2010^61^	Magne 2008^79^	Brunser 1989^67^	Bongers 2007^66^	Panigrahi 2008^82^	Augustina 2007^60^	Isolauri 2000^73^	Rautava 2009^83^	Decsi 2005^70^
Baldeon 2008^65^	Mah 2007^80^	Thibault 2004^91^	Euler 2005^71^	Karvonen 1999^96^	Alliet 2007^62^	Knol 2005^75^		Rinne 2005^85^
Chandra 2002^68^	Rinne 2006^86^		Kim 2007^74^	Karvonen 2001^97^	Bakker-Zierikzee 2005^63^	Nopchinda 2002^81^		Velaphi 2008^94^
Kuitunen 2009^76^	Saavedra 2004^88^		Rigo 2001^84^		Bakker-Zierikzee 2006^64^	Rivero 2004^87^		
Kukkonen 2007^77^	Sepp 1993^90^		Savino 2003^89^		Correa 2005^69^	Urao 1999^92^		
Kukkonen 2008^78^	Vendt 2006^95^				Hol 2008^72^	Van der Aa 2010^93^		

### Included studies

Summary of the included synbiotics, probiotics, prebiotics, and on-going studies are shown in Tables 2,3, 4, 5. All studies were conducted on healthy infants and used standard infant formula.

**Table 2:** Summary of 10 included probiotic studies

**Probiotic studies**	**Location**	**Inclusion criteria**	**Treatment used in study groups, n =**	**Treatment duration**	**Reported outcomes**
Brunser 2006^38^	Santiago - Chile	37 – 42 weeks gestation 3000 – 4200 g birth weight	1) Probiotic: *L Johnsonii La1* 10^8^ cfu/ g powder n=25	13 weeks	Average formula intake (ml/kg)
			2) Prebiotic: FOS 2g n= 32/L		Fecal excretion of bifidobacteria, Lctobacillus, Enterobacteria (Log10(CFU)/g stool)
			3) Breastfeeding n= 26		
			4) In Placebo group: Conventional infant formula no probiotic or prebiotic n= 33		
Chouraqui 2004^40^	France	Infants < 8 months	1) Probiotic: *B. lactis Strain Bb12 *1.5 × 10^6 ^cfu/g powder, n=46	148 days	Diarrhea, stools/day, Spitting, regurgitation
			2) In Placebo group: Conventional infant formula no probiotic or prebiotic, n=44		
Gibson 2009^44^	Adelaide -Australia	> 37 weeks gestation, birth weight 2500–4500 g,<10 days old	1) Probiotic group: *Bifibacterium lactis. *3.85 × 10^8^ cfu/g 100kcal, n= 72	7 months	Growth: Weight, length, head circumference
					Stool characteristics (data not shown)
			2) Placebo group: Conventional infant formula no probiotic, n=70		Stools, colic, spitting up, vomiting and restlessness
					Mean daily volume of formula intake
					GI infections, Respiratory infections
Haschke-Becher 2008^45^	Santiago - Chile	36 - 44 weeks gestation, birth weight > 2500 g at 16 weeks of age	1) Probiotic group:* Lactobacillus Johnsonii* 1 × 10^8^ cfu/g powder yielding 0.8 to 1.1 × 10^8^ cfu/ 200 mls formula, n= 17	4 weeks	Growth: Weight, length, Formula intake
Langhendries 1995^46^	Belgium, St Joseph-Montegnee-Rocourt	Healthy Full term infants	1) Probiotic group: *Bifibacterium Bifidum* 10^6^ cfu/g powder, n= 20	2 months	Bifidobacteria, Bacteriodes, Enterobacteria Log10 (CFU) / g of faeces
			2) Placebo group: conventional infant formula no probiotic, n= 20		
			3) Reference group: Human milk, n= 14		
Petschow 2005^48^	Iowa, USA	Healthy full term infants, weight >2500g, appropriate for gestational age (0–3 months of age)	1) Probiotic group:* Lactobacillus GG* 1 × 10^6^ cfu/g powder yielding 10^8^ cfu/day, n=15	7 day baseline, 14 days treatment period, 14 days follow up	Stool frequency, stool consistency
			2) Probiotic group: *Lactobacillus GG* 1 × 10^7^ cfu/g powder yielding 10^9^ cfu/day, n= 14		
			3) Probiotic group: *Lactobacillus GG* 1 × 10^8^ cfu/g powder yielding 10^10^ cfu/day, n= 15		
			4) Placebo group: Conventional infant formula no probiotic, n= 15		
Urban 2008^50^	Johanesburg South Africa	37 - 42 weeks gestation, 2500–4200 g birth weight, born to HIV+ mothers but infants tested HIV-	1) Probiotic group Acidified formula and *Bifidobacterium lactis* n= 29 (cfu/g powder is not specified)	4 months (119 days)	Growth: Males: Weight gain, length and head circumference
					Females: Weight gain, length and head circumference
			2) No probiotic group: Acidified formula no probiotic, n= 28		
			3) Placebo group: Conventional infant formula, (whey adapted formula), n= 28		
Weizman 2005^51^	Beer - Sheva Israel	> 38 weeks gestation, 4–10 months old	1) Probiotic group: *Bifidobacterium Lactis (BB-12)* 1 × 10^7^ cfu/g powder, n= 73	12 weeks	Episodes of diarrhea,
					Volume of feed / day
			2) Probiotic group: *Lactobacillus reuteri* 1 × 10^7^ cfu/g powder, n= 68		Episodes of respiratory illness, antibiotic use, clinic visits
			3) Placebo group: Conventional infant formula no probiotic, n= 60		
Weizman 2006^52^	Beer - Sheva Israel	> 38 weeks gestation, < 4 months (3–65 days of age)	1) Probiotic group: *Bifidobacterium Lactis (BB-12)* 1 × 10^7^ cfu/g powder yielding 2.2 × 10^8^ cfu/180 mls reconstituted formula, n= 20	4 weeks	Growth: Weight, length, head circumference (final percentiles)
					Stooling effort score, stooling consistency score
			2) Probiotic group: *Lactobacillus reuteri* 1 × 10^7^ cfu/g powder yielding 2.2 × 10^8^ cfu/180 mls reconstituted formula , n= 20		Daily crying score and daily crying episodes
					Formula volume (mls/kg)
			3) Placebo group: Conventional infant formula no probiotic, n=19		
Ziegler 2003^55^	Iowa USA	≥ 37 weeks gestation, Birth weight 2500g - 4500g (6–10 days of age)	1) No probiotic group: Reduced Protein formula no probiotic or prebiotic n=40	112 days	Growth: Males: Weight, length, Females, weight, length
			2) Probiotic group: Reduced protein formula*, Bifidobacterium lactis* 3.6 × 10^7^ cfu/g powder yielding 4.8 × 10^9^ cfu/L reconstituted formula , n= 40		Stool consistency
					Crying, colic (data not shown)
					Hospitalization, diarrhea, diarrhea (No. of episodes)
			3) Placebo group: Conventional infant formula, no probiotic, n=42		

**Table 3:** Summary of 12 included prebiotic studies

**Prebiotic studies**	**Location**	**Inclusion criteria**	**Treatment used in study groups, n =**	**Treatment duration**	**Reported outcomes**
Bettler 2006^37^	USA	<14 days postnatal age, birth weight and current weight between 10–90 percentiles for age	1) Prebiotic group: FOS 1.5 g/L n=72	12 weeks	Growth: Weight, length, Head circumference
			2) Prebiotic group: FOS 3.0 g/L n= 74		
			3) Placebo group: Conventional infant formula no prebiotic, n=66		
Bruzzese 2009^39^	Milan, Napoli, Verona Italy	37 to 42 weeks gestation, > 2500g birth weight, 4 to 6 months old	1) Prebiotic group: GOS, FOS (ratio 9:1) 0.4 g/100 ml n= 96	12 months	Growth, Weight, length. Stool consistency
			2) Placebo group: conventional formula with no prebiotic, N= 105		Infections: diarrhea episodes / child 12 months, episodes of acute diarrhea, episodes of URTI, antibiotic use
Costalos 2008^41^	Greece	Birth weight between 10th and 90th percentiles, no breastfeeding after age of 14 days	1) Prebiotic group: 90% G0S 10% LcFOS 0.4 g/100 ml n=70	6 weeks	Growth: Weight gain, length and head circumference gain
			2) Placebo group: Conventional formula no prebiotic n=−70		Stool frequency, consistency. GI Microflora: Bifidobacteria, E coli
Fanaro 2005^42^	Ferrara, Italy	Healthy full term infants, without antibiotic treatment	1) Prebiotic group: Acidic Oligosaccharides 0.2 g/dl, Maltodextrin 0.2 g/dl n= 16	6 weeks	Growth: Weight and length gain. Stool consistency
			2) Prebiotic group: Acidic Oligosaccharides 0.2 g/dl, Neutral GOS FOS 0.6 g/dl n= 15		Crying, regurgitation and vomiting episodes
			3) Placebo group: Maltodextrin at 8g/dl n=15		Gi Microflora
Fanaro 2008^43^	Ferrara, Turin Italy, Las Palmas, Seville Spain	Appropriate for gestational age, birth weight > 1500g, 4 to 6 months old	1) Prebiotic group: GOS 5 g/L n= 56	18 weeks	Growth: Weight, length
					Stool frequency, consistency
					GI microflora: Bifidobacteria, Lactobacilli, Bacteriodes,
					Clostridia, Enterobacteriacae
Moro 2002^35^ (Moro 2003, considered as one study)	Milan Italy	39 to 40 weeks gestational age	1) Prebiotic group: GOS, FOS 0.4 g/dl n=30	28 days	Growth: Weight and length gain
			2) Prebiotic group: GOS FOS 0.8 g/dl n= 27		Stool frequency, consistency
			3) Placebo group: Maltodextrin at 0.8g/dl n=33		Crying, regurgitation and vomiting
			4) Reference group: Breast milk n=15		Feeding volume
					GI microflora: Bifidobaceria, Lactobacilli
Moro 2005^47^	Italy	Healthy full term infants, appropriate for gestational age	1) Prebiotic group: GOS 0.8g/dl, n= 16	28 days	Growth: Weight, length gain
			2) Placebo group: Maltodextrin at 0. 8g/dl n=16		Feeding volume
					GI microflora
Moro 2006^37^ (Arslanoglu 2007, Arslanoglu 2008 considered as one study)	Milan Italy	37 - 42 weeks gestational age	1) Prebiotic group: ScGOS Lc FOS at 8g/L, n= 104	6 months	Growth: Weight gain, length gain, head circumference
			2) Placebo group: Maltodextrin at 8g/L, n=102		Stool frequency, consistency
					Crying, regurgitation and vomiting
					GI microflora: Bifidobacteria, Lactobacilli
					Infectious episodes: Overall infections, URTI, Otis Media, GI infections, UTI, antibiotic use
Schmelzle 2003^49^	Griefswald Germany	37 to 42 weeks gestational age, birth weight between 10 to 90 percentiles, exclusive formula feeding by age 14 days old	1) Prebiotic group: 90% GOS, 10% FOS 0.8/100ml n=76	12 weeks	Growth: Males - Weight gain, length gain, head circumference
			2) Placebo group: Conventional infant formula, no prebiotic, n=78		Females - Growth: Weight gain, length gain, head circumference
					Volume of feed (formula)
					GI microflora: Bifidobacteria
Xiao-Ming 2004^53^	Nanjing China	Healthy full term infants	1) Prebiotic group: Galactooligosaccharide 0.24 g/ dl n=69	6 months	GI Microflora: Bifidobacteria, Lactobacilli, E coli
			2) Prebiotic formula with Human milk n= 124		
			3) Placebo group: Conventional infant formula, no prebiotic, n=52		
			4) Reference group: Human milk n= 26		
Xiao-Ming 2008^54^	Nanjing China	> 38 weeks gestation, Birth weight > 3kg	1) Prebiotic group 1: Galactooligosaccharide 0.24 g/ 100 ml n=37	3 months	Growth: Weight gain, length gain
					Stool consistency
			2) Prebiotic group 2: Prebiotic formula with Human milk n= 58		Crying, regurgitation and vomiting scores
					Volume of feed
			3) Placebo group: Conventional infant formula, no prebiotic, n=45		GI Microflora: Bifidobacteria, Lactobacilli, E coli
			4) Reference group: Human milk n= 24		
Ziegler 2007^56^	USA	> 37 weeks gestation, Birth weight 2500g, solely formula fed	1) Prebiotic group 1: Polydextrose, Galactooligosaccharide n=58	120 days	Growth: Weight gain, length gain, head circumference
					Stool frequency, consistency
			2) Prebiotic group 2: Polydextrose, Galactooligosaccharide, Lactulose n= 48		Intolerance to formula: Vomiting, diarrhea, excessive spitting, colic
			3) Placebo group: Conventional infant formula, no prebiotic, n=58		

**Table 4:** Summary of 3 included synbiotic studies

**Probiotic studies**	**Location**	**Inclusion criteria**	**Treatment used in study groups, n =**	**Treatment duration**	**Reported Outcomes**
Chouraqui 2008^24^	France (Marseille)	37 – 42 weeks, gestation, ≤ 14 days singletons, 2500 – 4500g birth weight	1) Probiotic group: *Bifibacterium Longum *BL999 1.29 × 10^8^ cfu/100 ml formula, L.Rhamnosus 6.45 × 10^8 ^cfu/100 ml formula, n=60	4 months, observation: 16 – 52 weeks	Growth: Length, Head circumference Stool frequency, consistency, Incidence of diarrhea during treatment period Frequency of infections
			2) Synbiotic group 1: *Bifibacterium.Longum *BL999 1.29 × 10^8^ cfu/100 ml, L Rhamnosus 6.45 × 108 cfu/100 ml, 90% GOS, 10% ScFOS 0.4 g/100 ml n=54		
			3) Synbiotic group 2: *Bifibacterium Longum *BL999 2.58 × 10^8^ cfu/100 ml, LParacasei 2.58 × 10^8^ cfu/100 ml, 90% GOS, 10% ScFOS 0.4 g/100 ml, n=60		
			4) Placebo group: Conventional infant formula no probiotic or prebiotic, n=53		
Puccio 2007^25^	Palermo Italy	Healthy Full term infants with gestational age 39 weeks	1) Synbiotic group: *Bifibacterium Longum* BL 999 2 × 10^7^ Cfu/g powder, GOS 90% FOS 10% at 4g/L, n=42, n=67	112 days	Growth: Weight, length, head circumference
					Stool frequency (evacuations/day)
			2) Conventional infant formula no synbiotic, n=55		
					Crying, restlessness, colic, spitting and vomiting
					Volume of feed tolerated
					Frequency of respiratory tract infections
Vlieger 2009^21^	Niewegein, Netherlands	Healthy Full term infants with gestational age > 37 weeks, < 7 days, formula fed	1) Synbiotic group: *Bifibacterium animalis ssp Lacti*s 1 × 10^7^ Cfu/g powder, *Lactobacillusn paracase*i 1 × 107 Cfu/g powder, GOS 0.24 g/100 ml, n=67	6 months	Growth: Weight, length, head circumference
			2) Placebo group: Prebiotic infant formula GOS 0.24 g/100 ml, n=59		

**Table 5:** Summary of 3 on-going studies

**On-going studies**	**Location**	**Inclusion criteria**	**Treatment used in study groups, n =**	**Outcomes, Estimated date of completion**
Cabana 2010^57^	USA	>37 weeks gestation, birth weight >2500 g and < 4500 g, 14±3 days of age on enrollment, singleton birth, non- breastfed, not received solid foods.	1) Study group 1: Test starter infant formula	Primary: Weight gain (g/day) at 14 to 112 days of life (4 months)
			2) Study group 2: Test starter infant formula with synbiotics	
			3) Control /placebo group: Standard formula	Secondary: Tolerance, morbidity, protein status, metabolic markers December 2011
Zegerman 2009^58^	Israel	37th and 42 week gestation, birth weight > 2500 g, recruitment age: 0–28 days, non-breastfed	1) Study group 1: Dietary Supplement: probiotic microorganism and/or prebiotic	Primary: weight, length, head circumference
			2) Dietary Supplement: probiotic microorganism and/or prebiotic	Secondary: Microbiology August 2012
			3) Dietary Supplement: probiotic microorganism and /or prebiotic	
Ye 2010^59^	Singapore	> 37 weeks to < 42 weeks gestation, singleton birth. Age at enrolment < 14 days old	1) Study group 1: Standard infant formula with prebiotics	Primary: Mean Weight gain
			2) Study group: Infant formula with synbiotics	Secondary: Digestive tolerance December 2011

Synbiotic studies: Three studies (N = 475) used various synbiotic (probiotic and prebiotic) combinations [21,24,25]. Two studies [21,24] used a probiotic combination of *Bifidobacterium longum BL999* with *Lactobacillus rhamnosus; Bifidobacterium animalis ssp lactis* with *Lactobacillus paracasei.* One study [25] used *Bifidobacterium longum* alone. Dosage varied from 1 × 10^7^ to 2 × 10^7^ cfu/g powder to 1.28 × 10^8^ to 2.5 × 10^8^ cfu/100 ml. The prebiotics used were a combination of 90% GOS 10% FOS [24,25] or GOS alone [21]. The prebiotic doses ranged from 0.24 g to 0.4 g/100ml. Treatment duration varied from 4 months to 6 months. The synbiotic studies were conducted in France, Italy and Netherlands. None of the synbiotic studies reported data on volume of feed tolerated, hospitalization, changes in GI microflora and immune response.

Probiotic studies: Ten probiotic studies (N = 933) were included. One study [55] used a reduced protein infant formula and one study [50] used an acidified formula given to healthy infants born to HIV positive mothers. The most widely studied probiotics were *Bifidobacterium lactis (BB-12)* which was administered alone [40,44,46,50-52]. Other probiotic strains used were *Lactobacillus reuteri* and *Bifidobacterium bifidum*. Doses ranged widely. For Bifidobacteria: 1.5 x 10^6^ to 3.85 x 10^8^ cfu/g powder and Lactobacillus: 1 x 10^6^ to 1 x 10^8^ cfu/g powder. Treatment duration varied from 14 days to 7 months. The probiotic studies were conducted in Australia (Adelaide), Belgium, Chile (Santiago), France, Israel (Beersheva), South Africa (Johannesburg) and USA (Iowa). None of the probiotic studies reported data on immune response.

Prebiotic studies: Twelve prebiotic studies (N = 1563) were included. The studied prebiotics were FOS [37], GOS [43,47,53,54], acidic oligosaccharide [42] or a mixture of GOS and FOS [32,35,39,41,49]. Two studies used long chain FOS [32,41]. One study used poly dextrose with GOS [56]. The doses ranged from 0.15 g to 0.8 g/100 ml. Treatment duration ranged from 28 days to 12 months. The prebiotic studies were conducted in China (Nanjing), Greece, Germany (Griefswald), Italy (Ferrara, Milan, Turin, Verona), Spain (Los Palmas, Seville) and USA (Iowa). None of the prebiotic studies reported data on hospitalisation and immune response.

### Risk of bias

**Figure 3:**
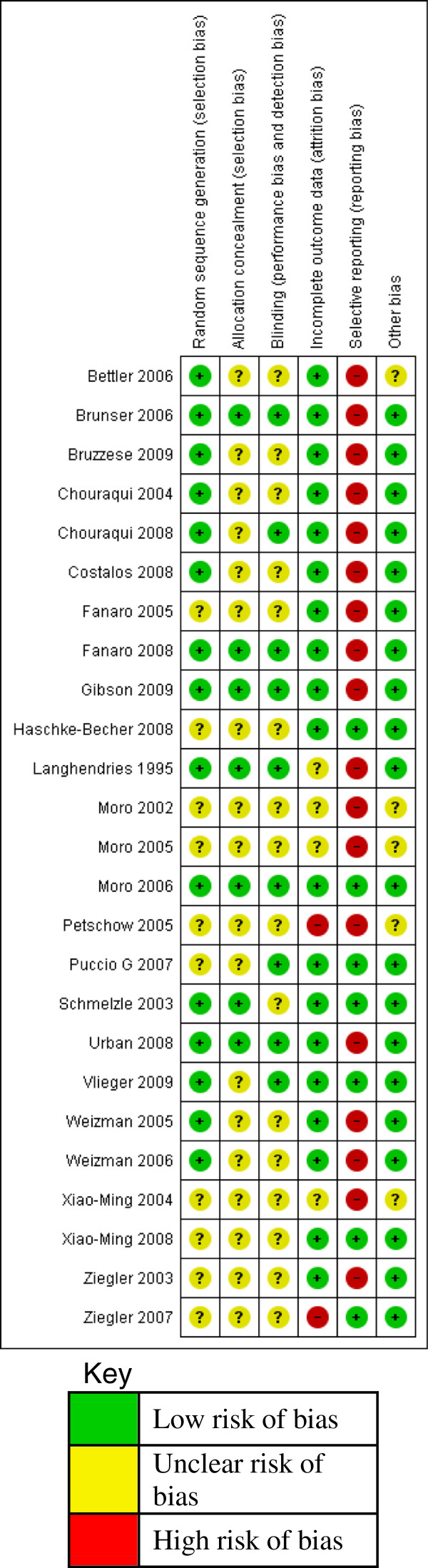
Methodological qualities of included studies.

The risk of bias of the included studies was assessed across six domains using guidelines from the Cochrane Handbook for Systematic Reviews of Interventions (Higgins 2008). See Figure 3.

#### Random sequence generation

Fifteen trials described clearly the methods used for random sequence generation [21,24,32,37,41,43,44,46,49-52]. Random sequence generation was done through computer randomization [21,37,38,43,44,50-52], random number tables [39,46] or block randomization [32,40,41]. The method used for random sequence generation was not clearly described in 10 studies [25,35,42,45,47,48,53-56].

#### Allocation concealment

In seven trials, treatment allocation was adequately concealed [32,38,42,44,46,49,50]. Allocation concealment was adequate due to central allocation using a computer [38], use of sealed envelopes [43,44,49], pre – coded or colour coded containers [32,50] and use of independent staff outside of study [46]. In the rest of the 18 studies, allocation concealment was not clearly demonstrated or described [21,24,25,35,37,39-42,45,47,48,51-56].

#### Blinding

Adequate blinding of study participants, care providers and assessors was done in 9 trials. Blinding was ensured by using pre-coded or colour coded formula tins [21,24,25,32,38,43,44,46,50]. In the other 16 trials, there was not enough information given on the blinding method to make a judgement [35,37,39-42,45,47-49,51-56].

#### Incomplete outcome data

Reported outcome data was satisfactory for 19 studies. In 3 studies, there was no missing outcome data [38,40,54]. In 16 studies, missing outcome data was balanced across the intervention groups with similar reasons reported [21,24,25,32,37,39,41-45,49-52,55]. In 4 studies there was insufficient information given to permit a judgement [35,46,47,53]. In 2 studies there were no reasons given for missing data [48,56].

#### Selective reporting

In 7 studies, the pre-specified outcomes in the methods section were reported in the results section [21,25,32,45,49,54,56]. In 18 studies the pre-specified outcomes were not reported [24,35-44,46,48,50-55].

#### Other potential sources of bias

Nineteen studies appeared to be free from other potential sources of bias [21,24,25,32,38-46,49-52,54,56]. There was insufficient information given to permit a judgment in 6 studies [35,37,47,48,53,55].

### Effects of interventions

#### Synbiotics versus controls

Three studies (N = 475) investigated the effect of synbiotic administration versus no synbiotic or placebo (control group) [21,24,25].

### Primary outcomes

#### Growth parameters

(i) Weight gain

Only one study [24] reported weight gain in terms of grams per day (g/day). In this study, two types of synbiotics (Type 1 and Type 2) were evaluated and results for boys and girls were reported separately. The results of the two synbiotics were combined using the combined mean and pooled standard deviation. The calculated treatment effects showed that synbiotics failed to significantly increase weight gain for boys (MD 0.90, 95% CI: -1.95 to 3.75, n = 81) and girls (MD 0.90, 95% CI: -1.81 to 3.61, n = 86) compared to the controls.

One study [21] reported weight gain in terms of some score scale. A calculated treatment effect showed that synbiotics failed to significantly increase weight gain compared to controls (MD −0.07, 95% CI: -0.43 to 0.29, n = 79). Since the score scale can take negative values, the values of mean and standard deviation in this analysis do not necessarily imply that the data is skewed.

One study [25] reported weight gain (g/day) in terms of mean difference (MD) and 90% CI. These values were used in calculating the corresponding standard error (SE). The MD and SE were used in calculating the treatment effect (via the generic-inverse variance method in RevMan). Synbiotics again failed to significantly increase weight gain compared to controls (MD −1.09, 95% CI: -3.54 to 1.36, n= 97).

(ii) Length gain

**Figure 4:**
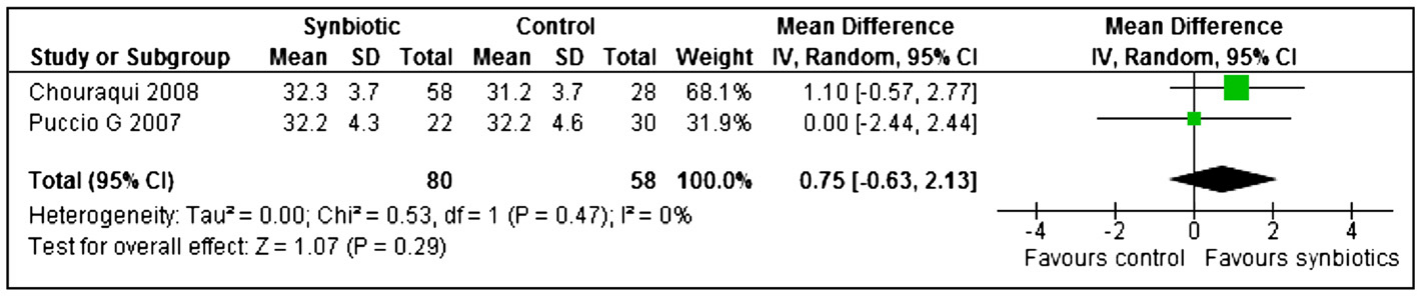
Synbiotics versus controls, Outcome: Length gain (mm/month) for girls.

Two studies [24,25] reported length gain in terms of millimetres per month (mm/month) for boys and girls separately. Results from these two studies were pooled in a meta-analysis but for Chouraqui 2008 [24] results for the two types of synbiotics were combined before meta-analysis. Results from the meta-analysis showed that synbiotics failed to significantly increase length gain compared to controls for both boys (MD 0.75, 95% CI: -0.66 to 2.17, n = 126) and girls (MD 0.75, 95% CI: -0.63 to 2.13, n = 138) [Figure 4. There was no significant heterogeneity detected between the two studies for boys (Chi^2^=0.50, df=1, p=0.48, I^2^=0%) and girls (Chi^2^=0.53, df=1, p=0.47, I^2^=0%).

One study [21] reported length gain in terms of some score scale. A calculated treatment effect showed that synbiotics failed to significantly increase length gain compared to controls (MD 0.01, 95% CI: -0.43 to 0.45, n = 79). Since the score scale can take negative values, the values of mean and standard deviation in this analysis do not necessarily imply that the data is skewed.

(iii) Head circumference gain

Two studies [24,25] reported head circumference gain in terms of mm/month for boys and girls separately. Results from these two studies were pooled in a meta-analysis but for Chouraqui 2008 [24] results for the two types of synbiotics were combined before meta-analysis. Results from the meta-analysis showed that synbiotics failed to significantly increase head circumference gain compared to controls for both boys. (MD −0.06, 95% CI: -0.96 to 0.85, n = 126) and girls (MD −0.05, 95% CI: -0.94 to 0.85, n = 138). There was no significant heterogeneity detected between the two studies for both boys (Chi^2^=0.64, df=1, p=0.43, I^2^=0%) and girls (Chi^2^=0.67, df=1, p=0.41, I^2^=0%).

One study [21] reported head circumference gain in terms of some score scale. A calculated treatment effect showed that synbiotics failed to significantly increase head circumference gain compared to controls (MD 0.01, 95% CI: -0.38 to 0.36, n = 79). Since the score scale can take negative values, the values of mean and standard deviation in this analysis do not necessarily imply that the data is skewed.

### Secondary outcomes

#### Tolerance to formula

(i) Stool frequency

**Figure 5:**
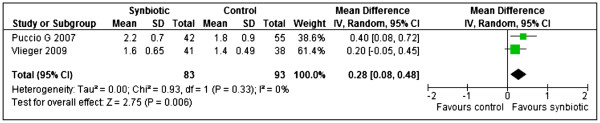
Synbiotics versus controls, outcome: Stool frequency (evacuations per day).

Two studies [21,25] reported stool frequency (evacuations per day) and their results were pooled in a meta-analysis. Synbiotics significantly increased stool frequency compared to the controls (MD 0.28, 95% CI: 0.08 to 0.48, n = 176) and there was no significant heterogeneity detected between the two trials (Chi^2^=0.93, df=1, p=0.33, I^2^=0%) [Figure 5.

One study [25] reported stool frequency (evacuations per day) but values for standard deviations were not given and as a result, no treatment effect could be calculated.

(ii) Stool consistency

One study [21] evaluated stool consistency using a consistency score (1=hard to 4=watery and loose) and a calculated treatment effect showed no significant difference between the synbiotic and control treated groups (MD 0.13, 95% CI: -0.15 to 0.41, n = 79).

One study [24] study reported that liquid stools occurred significantly more frequently in the synbiotic group compared to the control group (OR 3.17, 95% CI: 1.59 to 3.60, n = 66).

Puccio 2007 [25] reported that data on stool consistency showed no statistically significant differences between the two study groups (data not shown in study report).

(iii) Incidence of colic, spitting up / regurgitation, vomiting, crying

Data on frequency of crying, restlessness, colic, spitting and vomiting reported by Puccio 2007 [25] showed no statistically significant differences between the two study groups (data not shown in study report).

Results from Vlieger 2009 [21] showed no significant differences in the frequency of vomiting (RR 0.46, 95% CI: 0.12 to 1.72, n = 79) and colic (RR 2.50, 95% CI: 0.46 to 13.73, n = 79) between the two study groups. The same study showed no difference in crying (hours per day) between the two study groups (MD −0.10, 95% CI: -0.46 to 0.26, n = 79).

(iv) Average formula intake

One study [25] reported the mean daily intake of formula in a graph where no values could be retrieved.

One study [25] reported the mean daily intake of formula in a graph where no values could be retrieved.

#### Infections

(i) Infections

Puccio 2007 [25] reported data on frequency of respiratory tract infections but there were no significant differences between the synbiotic and control treated groups (RR 0.71, 95% CI: 0.31 to 1.59, n = 97).

Vlieger 2009 [21] reported the mean (SD) of upper respiratory tract infections and gastrointestinal infections (times per month) but no treatment effect could be calculated because the data was skewed (mean < SD).

(ii) Antibiotic intake

Vlieger 2009 [21] reported the mean (SD) of the use of antibiotics (times per month) but no treatment effect could be calculated because the data was skewed (mean < SD).

#### Probiotics versus controls

Ten studies (N = 933) investigated the effect of probiotic administration versus no probiotic (Control group) [38,40,44-46,48,50-52,55].

### Primary outcomes: growth parameters

(i) Weight gain

**Figure 6:**
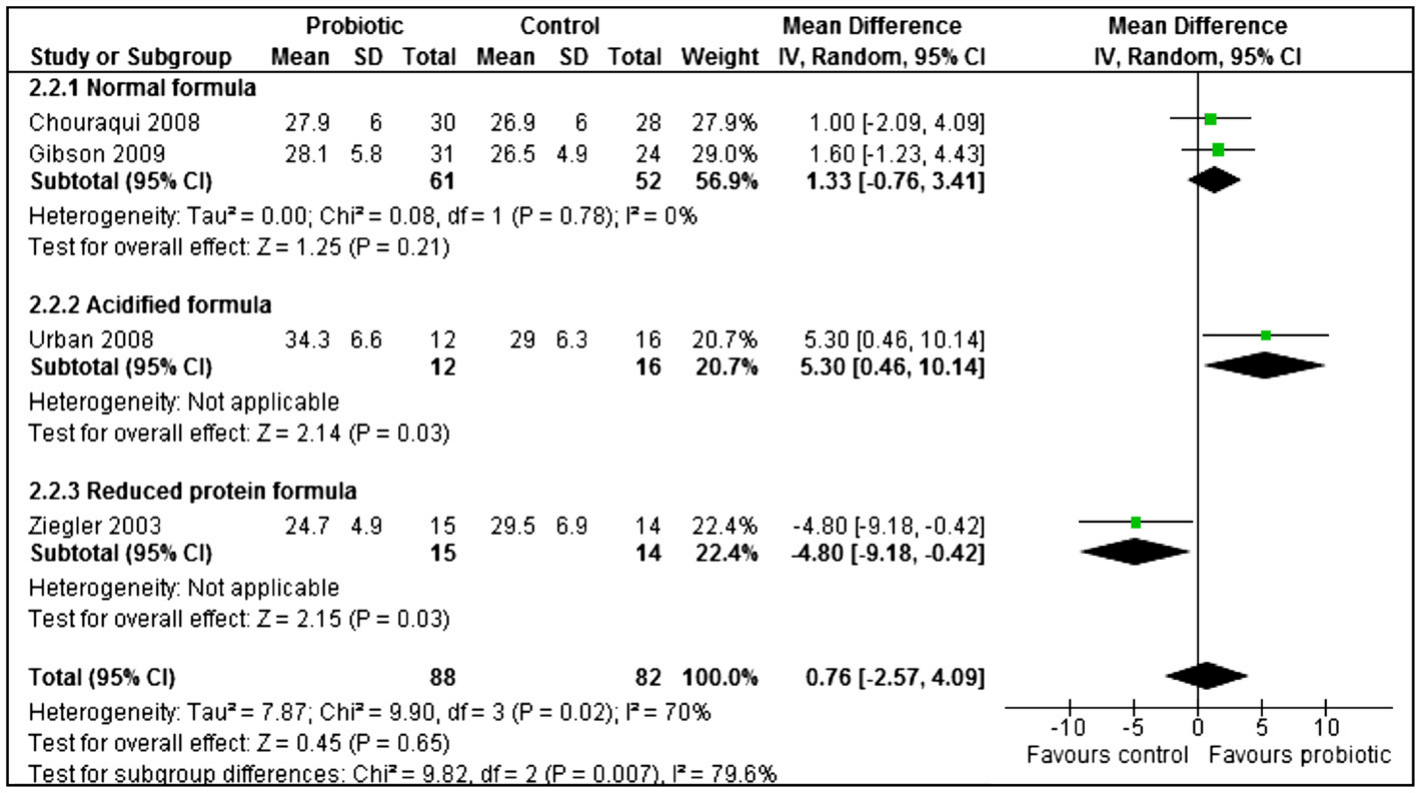
Probiotics versus controls, outcome: Weight gain (g/ day) for girls.

Four studies [24,44,50,55] reported weight gain (g/day) for boys and girls separately. The results from these four studies were pooled in meta-analyses separately for boys and girls. Probiotics failed to significantly increase weight gain compared to the controls for boys (MD 1.64, 95% CI: -0.36 to 3.64 n = 158), no statistically significant heterogeneity was detected between the studies for boys (Chi^2^=3.43, df=3, p=0.33, I^2^=13%). However, statistically significant heterogeneity was observed for girls (Chi^2^=9.90, df=3, p=0.02, I^2^=70%). An investigation of heterogeneity using subgroup analysis with respect to the type of formula (normal/ acidified/ reduced protein) yielded the following results. Two studies [24,44] showed that normal formula with probiotics failed to significantly increase weight gain compared to the controls for girls (MD 1.33, 95% CI: -0.76 to 3.41, n = 113) with no significant heterogeneity between the two studies (Chi^2^=0.08, df=1, p=0.78, I^2^=0%). Urban 2008 [50] showed that acidified formula with probiotics significantly increased weight gain in probiotic group compared to controls for girls (MD 5.30, 95% CI: 0.46 to 10.14, n = 28). Ziegler 2003 [55] showed that reduced protein formula with probiotics significantly reduced weight gain compared to controls for girls (MD −4.80, 95% CI: -9.18 to −0.42, n = 29) (Figure 6).

(ii) Length gain

Four studies [24,44,50,55] reported length gain for boys and girls separately. Two studies reported in terms of mm/month and two studies reported in terms of mm/day. The latter two studies results were converted to mm/month by multiplying both the mean and SD by 28, assuming a 4 week/ 28-day month. Results from these four studies were pooled in meta-analyses separately for boys and girls. Probiotics failed to significantly increase length gain compared to the controls for both boys (MD −0.37, 95% CI: -1.64 to 0.90, n = 158) and girls (MD 0.32, 95% CI: -0.81 to 1.45, n =165). No statistically significant heterogeneity was detected between the studies for both boys (Chi^2^=3.49, df=3, p=0.32, I^2^=14%) and girls (Chi^2^=2.94, df=3, p=0.40, I^2^=0%).

(iii) Head circumference gain

Three studies [24,44,50] reported length gain for boys and girls separately. Two studies reported in terms of mm/month and one study reported in terms of mm/day. The latter study's results were converted to mm/month by multiplying both the mean and SD by 28 (assuming a 4 week/ 28-day month). Probiotics failed to significantly increase head circumference gain compared to the controls for both boys (MD 0.76, 95% CI: -1.02 to 2.54, n = 125) and girls (MD 0.27, 95% CI: -0.70 to 1.23, n = 139). No statistically significant heterogeneity was detected between the studies for both boys (Chi^2^=3.87, df=2, p=0.14, I^2^=48%) and girls (Chi^2^=1.12, df=2, p=0.57, I^2^=0%).

### Secondary outcomes

#### Tolerance to formula

(i) Stool frequency

Two studies [40,48] reported stool frequency (evacuations per day) and meta-analysis of results from these studies showed that probiotics failed to significantly increase stool frequency compared to controls (MD 0.01, 95% CI: -0.44 to 0.46, n = 120). There was no significant heterogeneity between the studies (Chi^2^=0.19, df=1, p=0.66, I^2^=0%). Since Petschow 2005 [48] evaluated different probiotic dosages, the highest dosage was chosen for the analysis.

(ii) Stool consistency

One study [48] reported stool consistency score (1–5: 1=hard, 2=formed, 3=soft, 4=loose, 5=watery). A calculated treatment effect showed that there was no difference in consistency score between the probiotic and control groups (MD 0.00, 95% CI: -0.33 to 0.33, n = 30).

Chouraqui 2008 [24] reported that liquid stools occurred significantly more frequently in the probiotic group compared to the control group (OR 2.79, 95% CI: 1.48 to 5.29, n = 64).

Ziegler 2003 [55] reported stool consistency in terms of mean (SD) separately for hard, formed, soft and liquid stools but no treatment effect was calculated because the data was skewed (mean < SD). Weizman 2006 [52] reported results for stool consistency score but again the data was skewed (mean < SD).

(iii) Episodes of diarrhoea

Ziegler 2003 and Weizman 2005 [52,55] reported episodes of diarrhoea in terms of mean (SD) but no meta-analysis was done because their results show that the data was skewed (mean < SD).

Chouraqui 2004 and Chouraqui 2008 [24,40] reported the frequency of diarrhoea but meta-analysis of their results showed no benefit from probiotic treatment compared to controls (RR 0.80, 95% CI: 0.46 to 1.38, n = 209). No statistically significant heterogeneity was detected between the studies (Chi^2^=0.61, df=1, p=0.44, I^2^=0%).

(iv) Incidence of colic, spitting up / regurgitation, vomiting, crying

Chouraqui 2004 [40] reported the number of infants having spitting or regurgitation and there was no difference observed between the probiotic and control groups (RR 0.80, 95% CI: 0.26 to 2.42, n = 90). Weizman 2006 [52] reported the daily crying episodes and there were significantly less crying episodes in favour of the control group (MD 0.60, 95% CI: 0.20 to 1.00, n = 59). The results from the two probiotic groups were combined before meta-analysis. Gibson 2009 [44] reported that stools, colic, spitting up, vomiting, restlessness occurred at similar frequencies in the two groups (data not shown in report). Ziegler 2003 [55] reported that that there was no significant formula effects on crying and colic (data not shown in report).

(v) Average formula intake

One study [38] reported the average formula intake (ml/kg body-weight /day) and the calculated treatment effect showed no differences between the probiotic and control groups (MD 5.00, 95% CI: -12.60 to 22.60, n = 58). Two studies [44,51] reported the average formula intake (ml/day) and meta-analysis showed that infants in the probiotic group had a significantly higher formula intake compared to the controls (MD 46.74, 95% CI: 23.93 to 69.54, n = 292). No statistically significant heterogeneity was detected between the studies (Chi^2^=0.45, df=1, p=0.50, I^2^=0%).

### Infections

(i) Infections

One study [44] reported the number of infants having respiratory infections and the calculated treatment effect showed no differences between the probiotic and control groups (RR 0.93, 95% CI: 0.74 to 1.17, n = 142). One study [51] reported episodes of respiratory illness in terms of mean (95% CI). The mean (95% CI) were used in calculating the SDs. However, no treatment effect was calculated because the data was skewed (mean < SD). One study [44] reported the number of infants having gastrointestinal infections and the calculated treatment effect showed no differences between the probiotic and control groups (RR 0.70, 95% CI: 0.45 to 1.11, n = 142).

(ii) Antibiotic intake

One study [51] reported prescription of antibiotics in terms of mean (95% CI). The mean (95% CI) were used in calculating the SDs. However, no treatment effect was calculated because the data was skewed (mean < SD).

### Hospitalization

Only one study [55] reported hospitalization but no treatment effect was calculated because the data was skewed (mean < SD)

### Changes in gastrointestinal microflora

(i) Bifidobacteria

**Figure 7:**
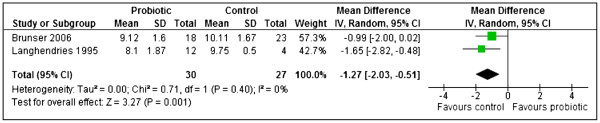
Probiotics versus controls, outcome: Bifidobacteria -log10(CFU) per gram of stool.

Two studies [38,46] reported results for bifidobacteria measured as log10 (CFU) per gram stool. A meta-analysis showed that the control group had significantly increased counts of bifidobacteria compared to probiotic group. (MD −1.27, 95% CI: -2.03 to −0.51, n = 57). No statistically significant heterogeneity was detected between the studies (Chi^2^=0.71, df=1, p=0.40, I^2^=0%) [Figure 7.

(ii) Lactobacillus

Only one study [38] reported results for lactobacillus, measured as log10 (cfu) per gram stool and the calculated treatment effect showed that probiotics failed to increase the counts of Lactobacillus compared to the controls (MD 0.22, 95% CI: -0.72 to 1.16, n = 41).

### Pathogens

(iii) Enterobacteria

Two studies [38,46] reported results for enterobacteria measured as log10 (cfu) per gram stool and meta-analysis showed that probiotics significantly reduced counts of Enterobacteria compared to the controls (MD −0.61, 95% CI: -1.20 to −0.03, n = 51). No statistically significant heterogeneity was detected between the studies (Chi^2^=0.62, df=1, p=0.43, I^2^=0%).

(iv) Bacteriodes

Two studies [38,46] reported results for bacteriodes measured as log10 (cfu) per gram stool and meta-analysis showed that probiotics failed to significantly reduce counts of Bacteriodes compared to the controls (MD −0.11, 95% CI: -1.01 to 0.78, n = 51). No statistically significant heterogeneity was detected between the studies (Chi^2^=0.95, df=1, p=0.33, I^2^=0%).

#### Prebiotics versus controls

Twelve studies (N = 1563) investigated the effect of prebiotic administration versus placebo or no prebiotic in formula (Control group) [32,35,37,39,41-43,47,49,53,54,56].

### Primary outcomes: growth parameters

(i) Weight gain

**Figure 8:**
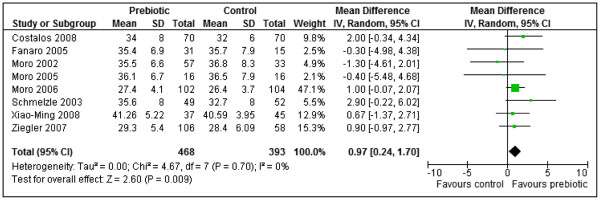
Prebiotics versus controls, outcome: weight gain (g/day).

Eight studies [32,35,41,42,47,49,54,56] reported weight gain (g/day) and meta-analysis of their results showed that prebiotics significantly increased weight gain compared to the controls (MD 0.97, 95% CI: 0.24 to 1.70, n = 861). No statistically significant heterogeneity was detected between the studies (Chi^2^=4.67, df=7, p=0.70, I^2^=0%). Three studies [35,42,56] evaluated different types of prebiotics (acidic oligosaccharides with maltodextrin or neutral GOS FOS, GOS FOS, GOS with polydextrose alone or with lactulose). The results for the prebiotics in each of these studies were combined before meta-analysis using combined mean and pooled standard deviation (Figure 8).

(ii) Length gain

Seven studies [32,35,41,42,47,49,54] reported length gain either as cm/week or in units that were converted to cm/week. Meta-analysis of their results showed that prebiotics failed to significantly increase length gain compared to the controls (MD 0.01, 95% CI: -0.01 to 0.04, n = 697). No statistically significant heterogeneity was detected between the studies (Chi2=5.05, df=6, p=0.54, I^2^=0%). Two studies [35,42] each evaluated different types of prebiotics (Acidic oligosaccharides 0.2 g/dl with maltodextrin, acidic oligosaccharides 0.2 g/dl with neutral GOS FOS 0.6 g/dl; GOS, FOS 0.4 g/dl and GOS FOS 0.8 g/dl). The results for the prebiotics in each of these studies were combined before meta-analysis using combined mean and pooled standard deviation.

(iii) Head circumference gain

Three studies [32,41,49] reported head circumference gain either as cm/week or in units that were converted to cm/week. Meta-analysis of their results showed that prebiotics failed to significantly increase head circumference gain compared to the controls (MD −0.01, 95% CI: -0.02 to 0.00, n = 438). No statistically significant heterogeneity was detected between the studies (Chi^2^=2.18, df=2, p=0.34, I^2^=8%).

Results from Ziegler 2007 [56] were not used because they reported head circumference gain only at 30 days and not at the end of treatment period which was 120 days. (All other studies reported results for end of treatment period).

### Secondary outcomes

#### Tolerance to formula

(i) Stool frequency

Four studies [32,35,43,56] reported stool frequency (evacuations per day) and meta-analysis of their results showed that prebiotics significantly increased stool frequency compared to the controls (MD 0.18, 95% CI: 0.06 to 0.30, n = 539). No statistically significant heterogeneity was detected between the studies (Chi^2^=2.97, df=3, p=0.40, I^2^=0%). Two studies [35,56] each evaluated different types of prebiotics (GOS FOS; GOS with polydextrose alone or with lactulose). The results for the prebiotics in each of these studies were combined before meta-analysis using combined mean and pooled standard deviation.

Costalos 2008 [41] reported the median (range) of stool frequency (Table 6).

**Table 6:** Stool characteristics

**Costalos 2008**^**41**^**: Median (range) stool characteristics**			
	**Prebiotics (n=70)**	**Controls (n=70)**	
**Stool frequency**	1.9 (1.2-2.1)	1.6 (1.1-1.9)	
**Stool consistency**	3 (2–3.5)	3.1 (2.5-3.5)	
**Moro 2002**^**35**^**: Median (IQR) Stool consistency score**			
	**Prebiotic1 (n=30)**	**Prebiotic2 (n=27)**	**Control (n=33)**
**Stool consistency score**	3 (1.5)	2.5 (0.75)	4 (1.5)

(ii) Stool consistency

Results from the two studies [32,42] using a 5-point scale (1=watery, 2=soft, 3=seedy, 4=formed, 5=hard) were pooled in a meta-analysis but due to significant heterogeneity detected between the two studies, their results are reported separately. Stools from the prebiotic group were significantly softer compared to controls for both Fanaro 2005 [42] (MD −1.20, 95% CI: -1.61 to −0.79, n = 46) and Moro 2006 [32] (MD −0.78, 95% CI: -1.00 to −0.56, n = 206). Fanaro 2005 [42] evaluated two types of prebiotics (acidic oligosaccharides with maltodextrin or neutral GOS FOS), the results for the prebiotics were combined before meta-analysis using combined mean and pooled standard deviation.

Fanaro 2008 [43] used an opposite 5 point scale (1=hard, 2=formed, 3=seedy, 4=soft, 5=watery) and reported the mean (SD) of area under the curve. A calculated treatment effect showed that stools from the prebiotic group were significantly softer compared to controls (MD 0.53, 95% CI: 0.31 to 0.75, n = 88).

Results from two studies [54,56] used a 4-point scale (1=watery, 2=soft, 3=seedy, 4=formed) were pooled in a meta-analysis but due to significant heterogeneity detected between the two studies, their results are reported separately. Stools from the prebiotic group were significantly softer compared to controls for both Xiao-Ming 2008 [54] (MD −0.65, 95% CI: -0.87 to −0.43, n = 82) and Ziegler 2007 [56] (MD −0.25, 95% CI: -0.44 to −0.06, n = 157). Ziegler 2007 [56] evaluated two types of prebiotics (GOS with polydextrose alone or with lactulose). The results for the prebiotics were combined before meta-analysis using combined mean and pooled standard deviation.

Costalos 2008 [41] reported the median (range) of stool consistency score (Table 6).

Moro 2002 [35] reported the median (IQR) of stool consistency score (Table 6).

(iii) Diarrhoea

Two studies [39,56] reported the number of infants having diarrhoea and a meta-analysis showed that prebiotics failed to significantly decrease the incidence of diarrhoea compared to the controls (RR 0.62, 95% CI: 0.19 to 1.99, n = 237). No statistically significant heterogeneity was detected between the studies (Chi^2^=1.65, df=1, p=0.20, I^2^=39%). Since Ziegler 2007 [56] evaluated two types of prebiotics (GOS with polydextrose alone or with lactulose), the number of events and totals for the prebiotics were summed before meta-analysis.

(iv) Incidence of colic, spitting up / regurgitation, vomiting, crying

Moro 2006 [32] reported no vomiting and very few infants crying but the number of infants experiencing regurgitation were significantly reduced in the prebiotic group compared to the control group (RR 0.11, 95% CI: 0.02 to 0.49, n = 206).

According to Xiao-Ming 2008 [54], there was no difference in crying score (MD 0.01, 95% CI: -0.00 to 0.02, n = 82), regurgitation score (MD −0.01, 95% CI: -0.27 to 0.25, n = 82), and vomiting score (MD −0.03, 95% CI: -0.21 to 0.15, n = 82) between the prebiotic and control groups. All scores were 3 point scores. Crying score: 1= practically not crying, 2 = crying in connection to feeding, 3 = crying independently from meals. Regurgitation score: 1 = no regurgitation, 2 = 1–2 regurgitations, 3 = > 2 regurgitations per day. Vomiting score: 1= no vomiting, 2 = 1 episode of vomiting, 3 = >1 episode of vomiting.

Ziegler 2007 [56] reported that none of the infants had colic; the numbers having excessive spitting were too few; vomiting was similar between the two groups (RR 1.12, 95% CI: 0.43 to 2.89, n = 32). The prebiotic results were summed for the two types before calculation of treatment effect.

Both Moro 2002 and Fanaro 2005 [35,42] reported no difference in the incidence of crying, regurgitation and vomiting episodes (data values not shown in study reports).

(v) Average formula intake

Five studies [35,38,47,49,54] reported formula intake (ml/kg body-weight/ day) and meta-analysis of their results showed statistically significant heterogeneity between the studies (Chi^2^=10.80, df=4, p=0.03, I^2^=63%,). Sensitivity analysis by removing the one study [49] showing significantly less formula intake for the prebiotics (MD −21.00, 95% CI: -31.86 to −10.14, n = 101) yielded no significant heterogeneity between the four remaining studies (Chi^2^=1.79, df=3, p=0.62, I^2^=0%) but no significant difference between the two groups (MD 0.31, 95% CI: -8.40 to 9.02, n = 269). The prebiotic results for the two types of prebiotics (GOS, FOS 0.4 g/dl, GOS FOS 0.8 g/dl) in Moro 2002 [35] were combined before meta-analysis using combined mean and pooled standard deviation.

### Infections

(i) Infections

According to Moro 2006 [32], prebiotics significantly reduced overall infections compared to the controls (RR 0.45, 95% CI: 0.29 to 0.69, n = 204). The number of infants having gastrointestinal infections, urinary tract infections (UTI) and otitis media were very few [32].

Two studies [32,39] reported the number of infants with upper respiratory tract infections (URTI) and their results were pooled in a meta-analysis. However, due to significant heterogeneity detected between the two studies (Chi^2^=7.69, df=1, p=0.006, I^2^=87%), their results are reported separately. Although Moro 2006 [32] showed that the prebiotic group significantly reduced the number of infants with URTI compared to the controls (RR 0.48, 95% CI: 0.27 to 0.84, n = 206), there was no difference between the two groups according to Bruzzese 2009 [39] (RR 1.07, 95% CI: 0.86 to 1.33, n = 203).

(ii) Antibiotic intake

According to Moro 2006 [32], prebiotics failed to significantly reduce antibiotic intake compared to the controls (RR 0.51, 95% CI: 0.26 to 1.00, n = 206).

### Changes in gastrointestinal microflora

(i) Bifidobacteria

**Figure 9:**
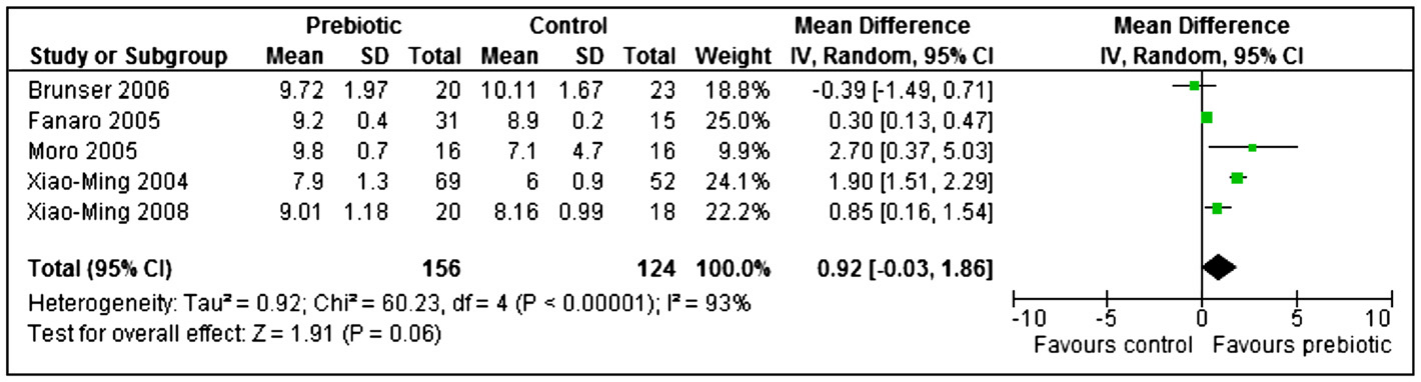
Prebiotics versus controls, outcome: Bifidobacteria -log10(CFU) per gram stool.

Five studies [38,42,47,53,54] (n = 280) reported Bifidobacteria (log10 CFU per gram stool) and their results were pooled in a meta-analysis. However, statistically significant heterogeneity was detected between the studies (Chi^2^=60.23, df=4, p < 0.00001, I^2^=93%). Heterogeneity persisted after conducting subgroup analysis with respect to duration of supplementation or dosage of treatment. The results for each study are therefore reported separately. Four studies showed that prebiotics significantly increased bifidobacteria: Fanaro 2005 [42] (MD 0.30, 95% CI: 0.13 to 0.47, n = 46); Moro 2005 [47] (MD 2.70, 95% CI: 0.37 to 5.03, n = 32); Xiao-Ming 2004 [53] (MD 1.90, 95% CI: 1.51 to 2.29, n = 121); Xiao-Ming 2008 [54] (MD 0.85, 95% CI: 0.16 to 1.54, n = 38). The prebiotic results for the two types of prebiotics (acidic oligosaccharides with maltodextrin or neutral GOS FOS) in Fanaro 2005 [42] were combined before meta-analysis using combined mean and pooled SD. However, Brunser 2006 [38] showed no significant difference in the number of bifidobacteria between the two groups (MD −0.39, 95% CI: -1.49 to 0.71, n = 43) [Figure 9.

Four studies reported their results in median; therefore no conclusions could be made. Costalos 2008 [41] reported the median (range) of Bifidobacteria (log10 CFU per gram stool) as a percentage of total bacteria (Table 7). Three studies [32,35,43] reported the median (IQR) of Bifidobacteria (log10 CFU per gram stool) (Table 8).

**Table 7:** Summary of findings table: Synbiotic studies

**Effects of infant formula containing Synbiotics on clinical outcomes in full term infants**					
**Patient or population: Full term infants, Settings: Multi-centre trials, Intervention: Infant formula with synbiotics, Comparison: Conventional infant formula**					
**Outcomes**	Illustrative comparative risks* (95% CI)		**Measure of effect (95% CI)**	**No of Participants (studies)**	**Quality of the evidence (GRADE)**
	**Assumed risk**	**Corresponding risk**			
	**Conventional formula**	**Infant formula with synbiotics**			
**Weight gain (g/day) for boys**	The mean (SD) weight gain (g/day) in control group was 30.9 (6.1)	Mean (SD) weight gain in synbiotic group was 31.8 (5.9)	MD (95% CI): 0.90 (−1.95 to 3.75)	81 (1 study)	⊕⊕⊝⊝
Follow-up: mean 4 months					**low**^1,2^
**Weight gain (g/day) for girls**	The mean (SD) weight gain (g/day) in control group was 26.9 (6)	Mean (SD) weight gain in synbiotic group was 27.8 (6)	MD (95% CI): 0.90 (−1.81 to 3.61)	86 (1 study)	⊕⊕⊝⊝
Follow-up: mean 4 months					**low**^3,4^
**Length gain (mm/mo) for boys**	The mean (SD) length gain (mm/month for boys in control group ranged from 32.6 (3.6) to 35.1 (4.4)	The mean length gain (mm/mo) for boys in the intervention groups was **0.75 higher** (0.66 lower to 2.17 higher)	MD (95% CI): 0.75 (−0.66 to 2.17)	120 (2 studies)	⊕⊕⊝⊝
Follow-up: mean 4 months					**low**^5,6,7^
**Length gain (mm/mo) for girls**	The mean length gain (mm/month) for girls in the control groups ranged from 31.2 (3.7) to 32.2 (4.6)	The mean length gain (mm/mo) for girls in the intervention groups was **0.75 higher** (0.63 lower to 2.13 higher)	MD (95% CI): 0.75 (−0.63 to 2.13)	138 (2 studies)	⊕⊕⊝⊝
Follow-up: mean 4 months					**low**^8,9,10^
**Head circumference gain (mm/mo) for boys**	The mean head circumference gain (mm/month) for boys in the control groups ranged from 17.4 (2.9) to 18.4 (2.3)	The mean head circumference gain (mm/mo) for boys in the intervention groups was **0.06 lower** (0.96 lower to 0.85 higher)	MD (95% CI): -0.06 (−0.96 to −0.85)	126 (2 studies)	⊕⊕⊝⊝
Follow-up: 4 to 6 months					
**Head circumference gain (mm/mo) for girls**	The mean head circumference gain (mm/month) for girls in the control groups ranged from 15.5 (3) to 16.7 (2.4)	The mean head circumference gain (mm/mo) for girls in the intervention groups was **0.05 lower** (0.94 lower to 0.85 higher)	MD (95% CI): -0.05 (−0.94 to 0.85)	138 (2 studies)	⊕⊕⊝⊝
Follow-up: 4 to 6 months					**low**^13,14^
**Stool frequency (evacuations per day)**	The mean (SD) stool frequency (evacuations per day) in the control group ranged from 1.4 (0.49) to 1.8 (0.9)	The mean stool frequency (evacuations per day) in the intervention groups was **0.28 higher** (0.08 to 0.48 higher)	MD (95% CI): 0.28 (0.08 to 0.48)	176 (2 studies)	⊕⊕⊝⊝
Follow-up: 4 to 6 months					**low**^15,16^

*The basis for the **assumed risk **(e.g. the median control group risk across studies) is provided in footnotes. The **corresponding risk **(and its 95% confidence interval) is based on the assumed risk in the comparison group and the **measure of effect **of the intervention (and its 95% CI).**CI: **Confidence interval, MD: Mean Difference.

GRADE Working Group grades of evidence. **High quality: **Further research is very unlikely to change our confidence in the estimate of effect. **Moderate quality: **Further research is likely to have an important impact on our confidence in the estimate of effect and may change the estimate. **Low quality:** Further research is very likely to have an important impact on our confidence in the estimate of effect and is likely to change the estimate. **Very low quality: **We are very uncertain about the estimate.

^1 ^Small sample size n=81, 95% CI includes no effect.

^2 ^Possible publication bias.

^3 ^Small sample size n=86, 95% CI includes no effect.

^4 ^Possible publication bias.

^5 ^Allocation concealment not described in 2 studies.

^6 ^Small sample size n=126.

^7 ^Possible Publication bias.

^8 ^Allocation concealment not described in 2 studies.

^9 ^Small sample size n=138.

^10 ^Possible Publication bias.

^11 ^Small sample size n=126.

^12 ^Possible publication bias.

^13 ^Small sample size n=138.

^14 ^Possible publication bias.

^15 ^Small sample size n=176.

^16 ^Possible publication bias.

**Table 8:** Gastrointestinal microflora

**Costalos 2008**^**41**^**: Median (range) as % of total bacteria**			
	**Prebiotics (n=70)**	**Controls (n=70)**	
**% Bifidobacteria**	39.69 (0–143.3)	14.87 (0–101)	
**% E.coli**	1.95 (0–69.32)	4.06 (0–59.31)	
**Fanaro 2008**^**43**^**: Median (IQR) microflora -log10(CFU) per gram stool**			
	**Prebiotics (n=56)**	**Controls (n=59)**	
**Bifidobacteria**	9.86 (8.99-10.18)	9.38 (8.35-9.90)	
**Lactobacilli**	4.62 (2–6.5)	4 (2–5.05)	
**Bacteriodes**	7.95 (6.64-9.6)	8.16 (6.3-9.04)	
**Clostridia**	4.3 (3–5.28)	4.29 (2.48-5.43)	
**Enterobacteria**	8.65 (8.12-9.13)	8.53 (7.96-9.01)	
**E. coli**	8.50 (7.9-8.99)	8.33 (7.59-8.83)	
**Moro 2002**^**35**^**: Median (IQR)**			
	**Prebiotic1 (n=30)**	**Prebiotic2 (n=27)**	**Control (n=33)**
**Bifidobacteria**	9.3 (1.6)	9.7 (0.8)	7.2 (4.9)
**Lactobacilli**	5.9 (1.5)	5.6 (2.1)	3.4 (1.8)
**Moro 2006**^**32**^**: Median (IQR) log10(CFU) per gram stool**			
	**Prebiotics (n=50)**	**Controls (n=44)**	
**Bifidobacteria**	10.28 (0.7)	8.65 (1.2)	
**Lactobacilli**	5.99 (3.6)	5.9 (2)	

(ii) Lactobacillus

Three studies [38,53,54] reported Lactobacillus (log10 CFU per gram stool) and meta-analysis of their results showed statistically significant heterogeneity between the studies (Chi^2^=26.44, df=2, p < 0.00001, I^2^=92%). Sensitivity analysis was done by removing the one study [38] that showed no difference between the two groups (MD −0.30, 95% CI: -1.08 to 0.48, n = 43). This yielded no significant heterogeneity (Chi^2^=0.33, df=1, p =0.57, I^2^=0%) between the remaining two studies. Meta-analysis showed that prebiotics significantly increased lactobacillus counts compared to the controls (MD 1.96, 95% CI: 1.58 to 2.34, n = 159).

Three studies reported their results in median; therefore no conclusions could be made. Fanaro 2008, Moro 2002 and Moro 2006 [32,35,43] reported the median (IQR) of Lactobacillus (log10 CFU per gram stool) (Table 8).

#### Pathogens

(iii) Enterobacteria

According to Brunser 2006 [38], there was no difference in the number of Enterobacteria counts between the prebiotic and control groups (MD −0.48, 95% CI: -1.88 to 0.22, n = 43). Fanaro 2008 [43] reported the median (IQR) of Enterobacteria (log10 CFU per gram stool) (Table 8).

(iv) Bacteriodes

According to Brunser 2006 [38], there was no difference in the number of Bacteriodes between the prebiotic and control groups (MD −0.35, 95% CI: -1.40 to 0.70, n = 43). Fanaro 2008 [43] reported the median (IQR) of Bacteriodes (log10 CFU per gram stool) (Table 8).

(v) E. coli

Two studies [53,54] reported E. coli (log10 CFU per gram stool) and their results were pooled in a meta-analysis. However, statistically significant heterogeneity was detected between the studies (Chi^2^=5.96, df=1, p=0.01, I^2^=83%). The results are therefore reported separately. Xiao-Ming 2004 [53] showed that prebiotics significantly reduced E. coli counts compared to the controls (MD −0.90, 95% CI: -1.29 to −0.51, n = 121) while Xiao-Ming 2008 [54] showed no significant difference between the two groups (MD 0.67, 95% CI: -0.53 to 1.87, n = 38).

Two studies reported their results in median; therefore no conclusions could be made. Costalos 2008 [41] reported the median (range) of E. coli (log10 CFU per gram stool) as a percentage of total bacteria (Table 8). Fanaro 2008 [43] reported the median (IQR) of E. coli and clostridia (log10 CFU per gram stool) (Table 8).

## Discussion

The objectives of this systematic review were to determine the effects of infant formula containing probiotics, prebiotics or both (synbiotics) on clinical outcomes in full term infants and to explore if synbiotics are superior over probiotics or prebiotics. Studies that used breast milk or mixed feeds (breast and infant formula or other types of milk) were excluded. All included RCTs evaluated either synbiotics, probiotics or prebiotics use in full term infants. The studies varied in enrolment criteria, sample size, intervention and treatment duration.

### Summary of main findings

#### Synbiotics

Addition of synbiotics to infant formula did not have any significant effect on growth (weight gain, length and head circumference). Synbiotics significantly increased stool frequency. However, two studies [21,25] reported no differences in stool consistency, while one study [24] reported an increase in liquid stools in synbiotic group. There were no significant differences between study groups on the incidence and frequency of colic, spitting up / regurgitation, crying, restlessness or vomiting. The effect of synbiotics on the volume of formula tolerated was not reported. Effect of synbiotics on frequency of infections was under reported. In one study [25], there were no differences in the frequency of infections between study groups, while in another study [21], the treatment effect could not be calculated or any conclusions made on the frequency of infections or antibiotic intake. Effects of synbiotics on hospitalization, GI microflora and immune response were not reported in any study therefore these parameters could not be evaluated.

Interpreting the effects of synbiotic supplementation of infant formula on clinical outcomes was difficult due to the limited number of studies. The synbiotic studies had short treatment duration (4 to 6 months) and treatment varied in all 3 studies. There was not enough evidence to state that synbiotics in infant formula have a significant effect on growth or lower the incidence of colic, spitting up / regurgitation, crying, restlessness. There is limited evidence that synbiotics do increase stool frequency and effects on stool consistency were inconclusive. There is not enough evidence to state that synbiotics reduce the risk of infections or decrease use of antibiotics. There is no data on the effects of synbiotics on GI microflora. The available data is very limited to draw reliable conclusions on the effects of synbiotics on clinical outcomes in formula fed infants.

#### Probiotics

A limited number of studies analyzed the effects of probiotic supplementation on growth by gender. These studies had small sample sizes and the follow-up periods were short. Addition of probiotics to infant formula did not have any significant effect on growth (weight gain, length gain or head circumference) in boys or girls. No study reported any weight loss. Probiotic infant formula was well tolerated. The limited available data shows that probiotics did not have any significant effect on stool frequency or consistency. Probiotic supplementation was not associated with fewer episodes of diarrhoea, a lower incidence of colic, spitting up / regurgitation, restlessness, vomiting. In one study [52] there were fewer crying episodes in the control group than probiotic group. Probiotic effects on infections, antibiotic use and length of hospitalization were inconclusive. Probiotic supplementation did result in a significantly higher formula intake compared to controls.

Effects of probiotic supplementation on intestinal microflora were conflicting. Probiotics failed to increase counts of bifidobacteria and lactobacillus. Probiotics significantly reduced counts of enterobacteria but failed to reduce counts of bacteriodes. None of the studies reported on immune response (CRP, IL-6), therefore the impact of probiotics on these parameters could not be evaluated. All 10 probiotic studies used various strains of bifidobacteria and lactobacillus with different doses. Treatment duration also varied from 14 days to 7 months. This confirms the ESPGHAN Committee on nutrition statement that there is a lack of published evidence on clinical benefits from long term use of probiotic containing infant formula [95]. Well designed long term follow – up RCTs using similar treatment regimens (same probiotics strains, dose and treatment duration) are needed to establish the effects of probiotics on healthy formula fed infants.

#### Prebiotics

Prebiotic addition to infant formula did have a significant effect on weight gain but had no significant effect on length and head circumference. None of the studies reported any weight loss. Prebiotic supplementation increased stool frequency but failed to improve stool consistency or decrease incidence of diarrhoea. Prebiotic supplementation did not reduce the incidence of spitting up / regurgitation, vomiting or crying (no study reported colic) or increased volume of formula tolerated. Prebiotic supplementation failed to significantly reduce upper respiratory infections. However, one study [32] did report a significant reduction in overall infections and antibiotic intake. Prebiotics supplementation failed to increase counts of bifidobacteria, lactobacillus or decrease the levels of pathogens (enterobacteria, bacteriodes, E – coli). None of the studies reported on hospitalization (days in hospital) and immune response (CRP, IL-6), therefore the impact of prebiotics on these parameters could not be evaluated.

Majority of the studies had a short treatment duration ranging from 28 days to 12 months. The prebiotic doses ranged from 0.15 g to 0.8 g/100 ml which did not exceed the level recommended by the European Committee on food in order to minimize intolerance and maximize the bifidogenic effect of the prebiotic.

#### Quality of the evidence and potential biases in the review process

We used guidelines from GRADE working group and GRADEpro 3.6 software to assess the quality of evidence in this review (Table 7, 9, 10). Overall the quality of evidence for primary outcomes is low, meaning that further research is very likely to have an important impact on our confidence in the estimate of effect and is likely to change the estimate. The quality of the evidence was compromised by: Imprecision (majority of studies had a small sample size ranging from 97 to 227 in the synbiotic studies, 54 to 201 in probiotic studies, 32 to 271 in the prebiotic studies); limitations in study design and execution (inadequate information was published to assess methodological quality of the study. Information was missing on sequence generation, allocation concealment, blinding, incomplete outcome data, selective reporting, free of other bias domains; Inconsistency of results); unexplained heterogeneity; use of different study preparations (types of synbiotic, probiotic, prebiotics) and different doses regimens were used and publication bias.

**Table 9:** Summary of findings table: probiotic studies

**Effects of infant formula containing Probiotics on clinical outcomes in full term infants**					
**Patient or population: Full term infants, Settings: Multi-centre trials (hospitals), Intervention: Infant formula with probiotics, Comparison: Conventional infant formula**					
**Outcomes**	Illustrative comparative risks* (95% CI)		**Measure of effect (95% CI)**	**No of Participants (studies)**	**Quality of the evidence (GRADE)**
	**Assumed risk**	**Corresponding risk**			
	**Conventional formula**	**Infant formula with probiotics**			
**Weight gain (g/day) for boys**	The mean (SD) weight gain (g/day) for boys in the control group ranged from 30.9 (6.1) to 32.8 (4.1)	The mean weight gain (g/day) for boys in the intervention groups was **1.64 higher** (0.36 lower to 3.64 higher)	MD (95% CI): 1.64 (−0.36 to 3.64)	158 (4 studies)	⊕⊕⊝⊝
Follow-up: 4 to 7 months					**low**^1,2^
**Weight gain (g/day) for girls**	The mean (SD) weight gain (g/day) for girls in the control group ranged from 26.5 (4.9) to 29 (6.3)	The mean weight gain (g/day) for girls in the intervention groups was **0.76 higher** (2.57 lower to 4.09 higher)	MD (95% CI): 0.76 (−2.57 to 4.09)	170 (4 studies)	⊕⊕⊝⊝
Follow-up: 4 to 7 months					**low**^3,4,5^
**Length gain (mm/month) for boys**	The mean (SD) length gain (mm/month) for boys in the control group ranged from 31.36 (4.48) to 37.3 (4.9)	The mean length gain (mm/month) for boys in the intervention groups was **0.37 lower** (1.64 lower to 0.9 higher)	MD (95% CI): -0.37 (−1.64 to 0.90)	158 (4 studies)	⊕⊕⊝⊝
Follow-up: 4 to 7 months					**low**^6,7^
**Length gain (mm/month) for girls**	The mean (SD) length gain (mm/month) for girls in the control group ranged from 28 (3.64) to 32 (4.6)	The mean length gain (mm/month) for girls in the intervention groups was **0.32 higher** (0.81 lower to 1.45 higher)	MD (95% CI): 0.32 (−0.81 to 1.45)	165 (4 studies)	⊕⊕⊝⊝
Follow-up: 4 to 7 months					**low**^8,9^
**Head circumference gain (mm/month) for boys**	The mean (SD) head circumference gain (mm/month) for boys in the control group ranged from 17.5 (3.4) to 35.28 (7)	The mean head circumference gain (mm/month) for boys in the intervention groups was **0.76 higher** (1.02 lower to 2.54 higher)	MD (95% CI): 0.76 (−1.02 to 2.54)	125 (3 studies)	⊕⊕⊝⊝
Follow-up: 4 to 7 months					**low**^10,11^
**Head circumference gain (mm/month) for girls**	The mean (SD) head circumference gain (mm/month) for girls in the control group ranged from16 (3) to 36.68 (15.4)	The mean head circumference gain (mm/month) for girls in the intervention groups was **0.27 higher** (0.7 lower to 1.23 higher)	MD (95% CI): 0.27 (−0.70 to 1.23)	139 (3 studies)	⊕⊕⊝⊝
Follow-up: 4 to 7 months					**low**^12,13^
**Bifidobacteria -log10(CFU) per gram of stool**	The mean (SD) bifidobacteria -log10(cfu) per gram of stool in the control group ranged 9.75 (0.5) to 10.11 (1.67)	The mean bifidobacteria -log10(cfu) per gram of stool in the intervention groups was **1.27 lower** (2.03 to 0.51 lower)	MD (95% CI): -1.27 (−2.03 to −0.51)	57 (2 studies)	⊕⊕⊝⊝
					**low**^14, 15^

*The basis for the **assumed risk **(e.g. the median control group risk across studies) is provided in footnotes. The **corresponding risk **(and its 95% confidence interval) is based on the assumed risk in the comparison group and the **measure of effect **of the intervention (and its 95% CI). CI: Confidence interval, CFU: colony forming units, MD: Mean Difference, RR: Risk ratio.

GRADE Working Group grades of evidence. **High quality:** Further research is very unlikely to change our confidence in the estimate of effect. **Moderate quality: **Further research is likely to have an important impact on our confidence in the estimate of effect and may change the estimate. **Low quality: **Further research is very likely to have an important impact on our confidence in the estimate of effect and is likely to change the estimate. **Very low quality: **We are very uncertain about the estimate.

^1 ^Small sample size n=158, 95% CI includes no effect.

^2 ^Possible publication bias.

^3 ^Unexplained heterogeneity).

^4 ^Small sample size n=170.

^5 ^Possible publication bias.

^6 ^Small sample size n=158, 95% CI includes no effect.

^7 ^Possible publication bias.

^8 ^Small sample size n=165, 95% CI includes no effect.

^9 ^Possible publication bias.

^10 ^Small sample size n=125, 95% CI includes no effect.

^11 ^Possible publication bias.

^12 ^Small sample size n=139.

^13 ^Possible publication bias.

^14 ^Small sample size n=57.

^15 ^Possible publication bias.

**Table 10:** Summary of findings table: prebiotic studies

**Effects of infant formula containing Prebiotics on clinical outcomes in full term infants**					
**Patient or population: Full term infants, Settings: Multi-centre trials, Intervention: Infant formula with prebiotics, Comparison: Conventional formula**					
**Outcomes**	Illustrative comparative risks* (95% CI)		**Measure of effect (95% CI)**	**No of Participants (studies)**	**Quality of the evidence (GRADE)**
	**Assumed risk**	**Corresponding risk**			
	**Conventional formula**	**Infant formula with prebiotics**			
**Weight gain (g/day)**	The mean (SD) weight gain (g/day) in the control group ranged from 26.4 (3.7) to 40.59 (3.95)	The mean weight gain (g/day) in the intervention groups was **0.97 higher** (0.24 to 1.7 higher)	MD (95% CI): 0.97 (0.24 to 1.70)	861 (8 studies)	⊕⊕⊝⊝
Follow-up: 1 to 6 months					**low**^1,2,3^
**Length gain (cm/week)**	The mean (SD) length gain (cm/week) in the control group ranged from 0.74 (0.1) to 0.96 (0.11)	The mean length gain (cm/week) in the intervention groups was **0.01 higher** (0.01 lower to 0.04 higher)	MD (95% CI): 0.01(−0.01 to 0.04)	697 (7 studies)	⊕⊕⊝⊝
Follow-up: 1 to 6 months					**low**^4,5,6^
**Head circumference gain (cm/ week)**	The mean (SD) head circumference gain (cm/ week) in the control group ranged from 0.34 (0.05) to 0.63 (0.1)	The mean head circumference gain (cm/ week) in the intervention groups was **0.01 lower** (0.02 lower to 0 higher)	MD (95% CI): -0.01 (−0.02 to 0.00)	438 (3 studies)	⊕⊕⊝⊝
Follow-up: 1.5 to 6 months					**low**^7,8^
**Stool frequency (evacuations per day)**	The mean (SD) stool frequency (evacuations per day) in the control group ranged from1.5 (0.6) to 2.4 (1.64)	The mean stool frequency (evacuations per day) in the intervention groups was **0.18 higher** (0.06 to 0.3 higher)	MD (95% CI): 0.18 (0.06 to 0.30)	579 (4 studies)	⊕⊕⊝⊝
Follow-up: 1 to 6 months					**low**^9,10^
**Diarrhea**	**Study population**		**RR 0.62** (0.19 to 1.99)	237 (2 studies)	⊕⊕⊝⊝
Follow-up: 4 to 12 months	**23 per 100**	**14 per 100** (4 to 46)			**low**^11,12^
	**Moderate**				
	**19 per 100**	**12 per 100** (4 to 38)			
**URTI**	**Study population**		**RR 0.74** (0.32 to 1.73)	409 (2 studies)	⊕⊕⊝⊝
Follow-up: 6 to 12 months	**45 per 100**	**33 per 100** (14 to 77)			**low**^13, 14, 15^
	**Moderate**				
	**44 per 100**	**33 per 100** (14 to 76)			
**Bifidobacteria -log10(CFU) per gram stool**	The mean(SD) bifidobacteria -log10(cfu) per gram stool in the control group ranged from 6(0.9) to 10.11 (1.67)	The mean bifidobacteria -log10(cfu) per gram stool in the intervention groups was **0.92 higher** (0.02 lower to 1.86 higher)	MD (95% CI): 0.92 (−0.03 to 1.86)	280 (5 studies)	⊕⊕⊝⊝
Follow-up: 1 to 6 months					**low**^16, 17, 18^
**Lactobacilli -log10(CFU) per gram stool**	The mean (SD) lactobacilli -log10 (cfu) per gram stool in the control group ranged from 3.95 (1.57) to 4.27 (2.02)	The mean lactobacilli -log10(cfu) per gram stool in the intervention groups was **1.12 higher** (0.44 lower to 2.67 higher)	MD (95% CI): 1.12 (−0.44 to 2.67)	202 (3 studies)	⊕⊕⊝⊝
Follow-up: 3 to 6 months					**low**^19,20,21^

*The basis for the **assumed risk **(e.g. the median control group risk across studies) is provided in footnotes. The **corresponding risk **(and its 95% confidence interval) is based on the assumed risk in the comparison group and the **measure of effect **of the intervention (and its 95% CI). CI: Confidence interval, CFU: Colony Forming Units, MD: Mean Difference, RR: Risk ratio.

GRADE Working Group grades of evidence: **High quality:** Further research is very unlikely to change our confidence in the estimate of effect. **Moderate quality: **Further research is likely to have an important impact on our confidence in the estimate of effect and may change the estimate. **Low quality: **Further research is very likely to have an important impact on our confidence in the estimate of effect and is likely to change the estimate. **Very low quality: **We are very uncertain about the estimate.

^1 ^Allocation concealment not clearly described in 6 studies.

^2 ^Blinding not clearly demonstrated or described in 7 studies.

^3 ^Possible publication bias.

^4 ^Allocation concealment not clearly demonstrated in 5 studies.

^5 ^Blinding not clearly demonstrated in 6 studies.

^6 ^Possible publication bias.

^7 ^Blinding not clearly described in 2 studies.

^8 ^Possible publication bias.

^9 ^Incomplete outcome data (with no reasons given for missing data) was present in 1 study.

^10 ^Possible publication bias.

^11 ^Small sample size n=237, 95% CI includes no effect.

^12 ^Possible publication bias.

^13 ^Unexplained heterogeneity.

^14 ^95% CI includes no effect.

^15 ^Possible publication bias.

^16 ^Unexplained heterogeneity.

^17 ^Small sample size n=280.

^18 ^Possible publication bias.

^19 ^Unexplained heterogeneity.

^20 ^Small sample size n=202.

^21 ^Possible publication bias.

At the conclusion of the review process and preparation of the manuscript (for this review), one on-going study [96] was recruiting, one study [97] was not yet recruiting, one study [98] was still on-going, no longer recruiting. Therefore data from these studies could not be included in this review. The reviewers used thorough comprehensive search strategies adopted for the available databases. All attempts were made to minimize publication bias. All steps of this review were conducted independently by the reviewers. Only randomised controlled studies were included in this review.

### Breast feeding statement

By conducting this review on exclusively formula fed infants, the authors do not seek to diminish the importance of breastfeeding and promote formula feeding. The reviewers acknowledge the importance of breastfeeding for infants. They support exclusive breastfeeding for 6 months, thereafter safe complementary feeding from 6 months of age with continued breastfeeding up to 2 years and beyond as per the global recommendations for optimal infant feeding of WHO and United Nations Children's Fund (UNICEF). This is because breastfeeding is the ideal feeding method for infants [99].

## Conclusion

There is not enough evidence to state that supplementation of term infant formula with synbiotics, probiotics or prebiotics does result in improved growth and clinical outcomes in full term infants. There is no data available to establish if synbiotics are superior to probiotics or prebiotics. Therefore this review does not support the routine supplementation of term infant formula with synbiotics, probiotics or prebiotics.

### Implications for practice

Probiotics: The limited evidence shows synbiotic or probiotic supplementation of infant formula did not have any adverse effects, significant impact on growth or clinical outcomes. All studies used different probiotic strains, the effects of one type of probiotic cannot be extrapolated to other types of probiotic bacteria. Prebiotic supplementation of infant formula also did not result in any adverse effects on infants. There are some clinical benefits such as improved weight gain and stool frequency.

### Implications for research

For clear recommendations to be made, well designed large RCTs with long term follow - up are required on exclusively formula fed term infants to investigate the effect of the same synbiotic combinations on clinical outcomes; the effect of the same probiotics (with similar doses and treatment duration) on clinical outcomes because available studies used different probiotic doses and treatment durations; the effect of the same prebiotics (with similar doses and treatment duration) on clinical outcomes because available studies used similar prebiotics with different doses and treatment duration; the effects of synbiotics, probiotics or prebiotics on clinical outcomes that have not been adequately addressed in previous studies; if synbiotics are superior to probiotics or prebiotics. Future RCTs should have treatment arms that include both synbiotics, probiotic and prebiotics.

## Abbreviations

Cfu: Colony Forming Units; CI: Confidence Interval; cm: Centimetres; ESPGHAN: European society for paediatric, gastroenterology, hepatology and nutrition; FOS: Fructooligosaccharide; g/day: Grams per day; GI: Gastrointestinal; GOS: Galactooligosaccharide; GRAS: Generally regarded as safe; IQR: Inter quartile range; IL-6: Interleukin – 6; MD: Mean difference; mm: millimetres; RCTs: Randomized controlled trials; RR: Risk ratio; SD: Standard deviation; UNICEF: United nations children's fund; UTI: Urinary tract infections; URTI: Upper respiratory tract infections; WHO: World Health Organisation.

## Competing interests

All reviewers declared no competing interests.

## Authors’ contributions

The reviewers contributed the following: MM: Developed review protocol (unpublished), selected RCTs, conducted data extraction, assessment of risk of bias in included studies, developed, edited and critically reviewed the manuscript. ML: Selected RCTs, conducted data extraction, assessment of risk of bias in included studies, critically reviewed the manuscript. AM: Conducted the statistical analysis, interpretation of results and critically reviewed the manuscript. TY: Assisted in designing the review and critically reviewed the manuscript. RB: Assisted in designing the review and critically reviewed the manuscript. All authors’ read and approved the final manuscript.

## Authors’ information

^1^Division of Human Nutrition, Faculty of Medicine and Health Sciences, Stellenbosch University, South Africa, ^2^Wits Reproductive Health & HIV Institute (WRHI), Faculty of Health Sciences, University of the

Witwatersrand, Johannesburg, South Africa, ^3^Centre for Evidence-Based Health Care, Faculty of Medicine and Health Sciences, Stellenbosch University, South Africa
